# What is the effectiveness of the support worker role for people with dementia and their carers? A systematic review

**DOI:** 10.1186/s12913-016-1531-2

**Published:** 2016-07-19

**Authors:** Dianne Goeman, Emma Renehan, Susan Koch

**Affiliations:** RDNS Institute, Royal District Nursing Service Ltd, 31 Alma Rd, St Kilda, VIC 3182 Australia

**Keywords:** Community dwelling people with dementia, Carers, Support workers

## Abstract

**Background:**

Dementia is progressive in nature and the associated functional decline inevitably leads to increasing dependence on others in areas of daily living. Models of support have been developed and implemented to assist with adjusting to living with memory loss and functional decline; to navigate the health and aged care system; and to access services. We undertook a systematic review of international literature on key worker type support roles to identify essential components and ascertain how the role can be best utilised to assist community-dwelling people with dementia and their carers. This review of support roles is the first to our knowledge to include both quantitative and qualitative studies and all models of support.

**Method:**

A systematic review of studies written in English and published between January 2003 and December 2014. Data sources were Medline, PsychInfo and CINAHL, internet, expert consultation and reference lists of included studies. After screening articles to ensure that they reported on a key worker type support role, involved carers and or people with dementia living at home and removing duplicates, eligible papers were appraised and evaluated.

**Results:**

Thirty six studies were eligible for inclusion in the review. Eligible studies were divided into type of support roles and study type. The heterogeneity of included studies and high risk of bias made a meta-analysis inappropriate and it was therefore difficult to draw overall conclusions. However, essential components shared across support worker models that demonstrated a positive impact on carer burden and improved quality of life included: long term intervention, face to face contact, individualised education and support based on needs, multi-disciplinary teams, collaborative input, health/clinical background of support workers, ongoing follow up and inter professional and inter-sectoral collaborations. There was a lack of studies assessing cost-effectiveness.

**Conclusions:**

Studies that include a high quality evaluation of holistic, tailored models of support that identify which components of support produce the most valuable outcomes to assist people with dementia and their carers and families to continue to live meaningful lives are needed. There is also a need for a cost effectiveness evaluation of support worker roles.

**Trial registration:**

PROSPERO international prospective register of systematic reviews: PROSPERO 2014 CRD42014013992.

**Electronic supplementary material:**

The online version of this article (doi:10.1186/s12913-016-1531-2) contains supplementary material, which is available to authorized users.

## Background

Dementia is progressive in nature and leads to a decline in cognitive abilities. In the early stages of the disease, people with dementia may experience difficulties undertaking routine tasks, such as driving, shopping and managing their finances. As the disease progresses there may also be difficulties with self-care, bathing, eating and communication [1]. As a consequence of these difficulties many people with dementia who live at home are supported by informal carers.

The World Health Organisation has estimated that over 46 million people worldwide are living with dementia and that this number will increase to 74 million by 2030 [2]. This rapid increase in prevalence of dementia is expected to pose a substantial challenge to health, aged care and social policy and substantially increase the number of informal care givers [2].

Providing support for a person with dementia can lead to a decline in physical and mental health and can also impact employment and education prospects, finances and participation in social and community life [3]. Disease progression also leads to increasing difficulty in continuing to meet the needs of a person with dementia in the home setting and as a result care has progressively shifted from the private to the public setting through the introduction of home based support services provided in the community [4].

Despite these formal services being available, the use of services by carers (including respite) is quite low [5]. This has been attributed to the services being difficult to navigate, not meeting carer/care recipient needs, and beliefs that service use would result in negative outcomes for the care recipient [5] The non-use of formal services has also been associated with high levels of depression among carers [5].

In order to address the difficulties and the stresses associated with living with cognitive impairment models of support to assist adjusting to living with memory loss; to navigate the health and aged care system and to access services and information have recently been implemented both in Australia and internationally [6]. Support worker type roles include case managers, care workers, counselling support workers and multi-team integrated care. In the United Kingdom there is also the admiral nurse role, that utilises a specialist mental health nurse, and in Australia the role of the key worker, designed to provide support to people with younger onset dementia.

Currently, there is a lack of high level evidence regarding the overall effectiveness of these support roles for people with dementia and their carers [7]. Previous systematic reviews of dementia support worker roles have focused on case management roles [8–14] with only one extending this to include other support models (integrated care and consumer directed care) [15]. Our comprehensive systematic review of the international literature of models of support for community-dwelling people with dementia and their carers aims to develop an evidence-informed national approach by health and aged care service providers, government and consumers to support people with dementia, their carer’s and families. Our extensive systematic review of the international and national academic literature of models of support for community-dwelling people with dementia and their carers is the first to our knowledge to include both quantitative and qualitative studies and all models of support.

## Methods

The review questions were:

1. What are the essential components of the key worker type model of support for people living with dementia and their carers
2. How can the role be best utilised to assist community-dwelling people with dementia and their carers?

### Data sources and search strategy

Literature indexed in the scientific databases MEDLINE, CINAHL and PsychINFO, was searched. Google Scholar was used to identify studies that did not appear in the scientific databases.

The search conducted in EBSCO MEDLINE, CINAHL and PSYCHOINFO used key words and subject headings limited to English language published between 2003 and December 2014. Subject headings included: (“Alzheimer disease” OR “Dementia” OR “Dementia, Multi-infarct” OR “Fronto-temporal Dementia” OR “Dementia, Vascular” OR “Lewy Body Disease”) OR (keywords “dementia” OR “Alzheimer’s”). Key words included: “key worker”, “link worker”, “support worker”, (“case management” as key word OR “Case management” as subject heading), “case manager”, (“nurse clinician” as key word OR“Nurse clinicians” as subject heading), “clinical nurse consultant”, “admiral nurse”, (“patient navigation” as key word OR “Patient Navigation” as subject heading), “navigator”, “nurse specialist” then all of these searches were combined with OR. Finally, the combined search of roles (i.e. key worker etc.) was added together with the combined search of dementia using AND to produce the final search.

### Inclusion and exclusion criteria

To ensure that our review was relevant to current practice we included research papers written in English language and published between January 2003 and December 2014. All study designs of articles that examined key worker type support roles for people with dementia living at home and carers of people with dementia living at home were appraised.

The key worker type support roles included were: case managers, care managers, support worker, admiral nurse, link worker, key workers, counselling roles and team based/multi-agency/integrated care roles.

#### Outcomes

Evaluation of key worker type roles; reduced carer burden; improved quality of life, improved symptom severity for people with dementia and reduced institutionalisation rates.

We excluded articles published prior to 2003 and not written in English. We also excluded articles that were case reports, editorials and opinion pieces rather than reports of an intervention or description of a support worker model.

#### Study selection process

All evaluations, descriptive and comparative studies of the utilisation or role of key worker type support models assisting community-dwelling people with dementia and their carers were screened independently by two authors. Initially, the title and abstract of the all indentified studies were screened for eligibility. An eligibility instrument was used to guide the decisions (see Additional file 1).

## Quantitative data

### Potential effect modifiers and reasons for heterogeneity

As the types of studies included in the systematic review were heterogeneous, and after consideration of the risk of bias, they were not suitable for inclusion in a meta-analysis. Therefore we undertook a comparison of the studies and their outcomes synthesising the data into tables according to types of support worker roles and study types.

### Quality assessment

One of the purposes of conducting research is to provide evidence of efficacy, however, not all evidence is considered equal [16]. Consequently, we considered the levels of evidence before summarising the information. Two authors independently appraised the quality of all included quantitative and qualitative articles. Where there were discrepancies in appraisal, papers were re-read by both assessing authors and consensus reached through discussion.

Acceptable levels of information were decided using the NHMRC Grade levels (see Table 1) and the Cochrane and CASP Risk of Bias Tools to guide decisions.

**Table 1:** Designation of Levels of Evidence

Designation of levels of evidence	
Level I	Evidence obtained from a systematic review of all relevant randomised controlled trials
Level II	Evidence obtained from at least one properly designed randomised controlled trial
Level III-1	Evidence obtained from well-designed pseudo-randomised controlled trials (alternate allocation or some other method)
Level III-2	Evidence obtained from comparative studies with concurrent controls and allocation not randomised (cohort studies), case-control studies, or interrupted time series with a control group
Level III-3	Evidence obtained from comparative studies with historical control, two or more single-arm studies, or interrupted time series without a parallel control group
Level IV	Evidence obtained from case series, either post-test or pre-test and post-test

### Risk of bias

To assess the presence/risk of bias of the studies we identified we used the Cochrane Risk of Bias assessment tool for RCTs and non-randomised or quasi-experimental studies. For observational studies, we adapted the Critical Appraisal Skills Programme (CASP) checklist for cohort studies and the CASP checklist for case control studies to determine risk of bias.

### Data extraction

All identified studies were screened for eligibility based on titles and abstracts using an eligibility assessment tool to determine if the study utilised or discussed a support worker type role, if the participants had dementia or cognitive decline or were carers of people with dementia or cognitive decline and whether the participants were community dwelling/living at home or were carers of community dwelling people with dementia or cognitive impairment (see Additional file 1).

#### Data synthesis and presentation

The selected studies/papers were categorised into type of role and study type. In most cases, but not all the studies/papers were mutually exclusive to their categories. Country of origin, year of study and whether the study was registered was recorded for each study/paper. Electronic PDF versions of all eligible studies were retrieved prior to undergoing a critical appraisal. No attempts were made to contact authors for additional information.

Duplicates were removed, and titles identified in the electronic search were read, to identify those that were relevant. Abstracts were reviewed, and where they were identified to meet the inclusion criteria, the full publication was obtained and assessed for eligibility.

Two researchers screened records for inclusion in the review using the GATE framework tool to undertake a critical appraisal of the quantitative studies [17] (see Additional file 1). The schedule derived from this framework considered: population, exposure and comparison groups, outcomes, time, results and applicability (generalisability, feasibility and relevance, applicability and potential harms [17].

#### Qualitative data

Qualitative studies exploring the experience of those involved in interventions and evaluating factors that shape the implementation of interventions have an important role in ensuring that systematic reviews are of maximum value to policy, practice and consumer decision making [18–20]. Therefore, we also included a synthesis of the qualitative evidence in this review.

As many of the studies/papers were qualitative Greenhalgh & Taylor’s paper [21] and Britten & Pope’s work on synthesising qualitative studies were drawn upon to appraise these qualitative studies [22]. This appraisal considered: if the paper described an important problem and if the authors answered the question they set out to, methodological approach (were methods appropriate, setting, participants, recruitment, aims, recruitment bias, researcher perspective, interview schedule design, data collection, recording and transcription, data analysis, validity and reliability, if the results were credible, conclusions and if these were justified and whether the findings were transferable to other settings (see Additional file 1).

The appraisal was undertaken by two independent members of the research team (DG and ER). Results of appraisals by the two independent researchers were compared and differences resolved through discussion and revisiting the criteria associated with each of the critical appraisal tools. Consensus was achieved in all cases.

#### Risk of bias

Qualitative studies were critiqued according to Greenhalgh & Taylor’s [21] and Britten & Pope’s [22] frameworks for assessing/synthesising qualitative studies. This process examined the context, theoretical approach, categories, concepts and interpretation of each study.

## Results

**Fig. 1:**
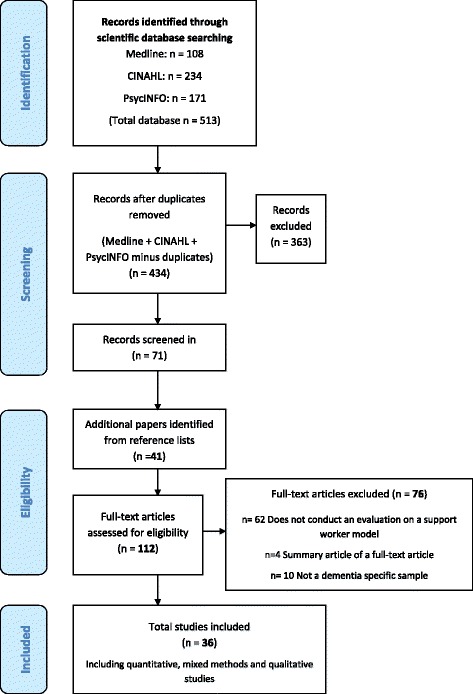
Prisma flow chart - Details of study flow

The search strategy identified 513 references (Fig. 1). After removal of duplicates 434 abstracts were examined for relevance and full text for 71 references were obtained for full screening. Hand-searching of reference lists of included articles yielded an additional 41 articles. In total 112 articles were assessed for eligibility, of which 36 articles were selected for data extraction and analysis.

### Study characteristics

Of the 36 included studies, 24 were Randomised Controlled Trials (RCTs), eight were qualitative, two were mixed method, one was a case report and one was a cohort study. The studies were conducted in the United States of America (*n* = 16), Europe (*n* = 5), United Kingdom (*n* = 8), Hong Kong (*n* = 3), Australia (*n* = 2), Canada (*n* = 1) and one was conducted across the United Kingdom, United States of America and Australia (*n* = 1).

The majority of studies evaluated counselling support roles. The rest evaluated support worker (including key worker, link worker, Admiral Nurses), case manager, team-based/multi-agency/integrated support roles, and care manager roles.

### Case manager roles

The seven studies whose interventions involved case manager roles covered a broad range of study designs. These included four RCTs, two mixed method studies and one qualitative study [23–29] (see Tables 2, 3, 4 and 5).

**Table 2:** Case Management RCT Outcomes

Study	Carer outcomes	Person with dementia outcomes
Chien and Lee 2008 [23]	• Burden • Quality of life • Social support • Access of Community Services	• Symptom severity • Institutionalisation rates
Chien and Lee 2011 [24]	• Burden • Quality of life • Social support • Access of Community Services	• Symptom severity • Institutionalisation rates
Jansen et al. 2011 [25]	• Sense of competence • Quality of life • Depressive symptoms • Burden	• Quality of life
Lam et al. 2010 [26]	• Burden • General health • Personal wellbeing	• Symptom severity • Depressive symptoms • Personal wellbeing

**Table 3:** Randomised Controlled Trials – Case Manager Roles – Level II evidence

Article	Sample	Intervention	Control	Outcome measures	Outcomes/results	
Chien and Lee 2008 [23] Hong Kong	*N* = 88 dyads Primary Caregivers and people with dementia.	• *N* = 44 Six-month Dementia education and support program for carers • Multi-disciplinary committee including psychiatrist, social worker, case nurse manager from each centre and researchers selected 25 intervention goals and objectives from the recommended dementia guidelines • Case management by a Nurse who received 32 h of formal training by researchers • Case manager: provided case management, coordinated all levels of family care according to the results of structured needs assessment, formulated a multidisciplinary education program for each family on effective dementia care, provided community support resources, and reviewed the program.	• *N* = 44 Routine Dementia care: pharmacotherapy and social and recreational activities, written educational material and six monthly-education sessions	• Caregiver burden – *Family Care giving Burden Inventory* • Quality of life – *WHO Quality of Life Scale* • Social support – *Six-item Social Support Questionnaire* • Symptom Severity *– Neuropsychiatric Inventory and MMSE* • Access of Community Services – *Family Support Services Index*	• No loss to follow-up • Significant reductions in the institutionalisation rate at 6 and 12 months • Significantly greater improvements in quality of life and burden in caregivers at 6 and 12 months • Significantly greater improvements in patients symptom severity at 6-months only • Significant reduction in family service utilisation at 12-months	Preliminary level II high quality evidence to support a 6 month dementia education and support management program for improving caregiver quality of life and burden and reducing institutionalisation rates
Chien and Lee 2011 [24] Hong Kong	*N* = 92 family members caring for a relative with dementia at home	• *N* = 46 • Six-month Dementia Family Care Programme – individualised education and support program for effective dementia care • Multi-disciplinary committee including psychiatrist, social worker, case nurse manager from each centre and researchers selected 25 intervention goals and objectives from the recommended dementia guidelines. • Case management by a Nurse who received 32 h of formal training by researchers • Case manager conducted weekly home visits, family health and educational needs assessment, education about dementia care and collaborated with caregivers to prioritise the problems and formulated an individualised education and support program for each family • Case manager guided each family using six step model: defining the problem, generation of alternatives, examining and evaluating each alternative, cognitive rehearsal of action plan, execution of plan as home work and evaluation of outcomes	• *N* = 46 • Routine family services including medical consultation, advice and referrals for financial aid and social welfare, education talks and social and recreational activities	• Caregiver burden – *Family Caregiving Burden Inventory* • Quality of life – *WHO Quality of Life Scale* • Social support – *Six-item Social Support Questionnaire* • Symptom Severity *– Neuropsychiatric Inventory and MMSE* • Access of Community Services – *Family Support Services Index*	• All 92 participant data included in follow-up • Statistically significant improvement in caregivers burden and quality of life over 18 months • Statistically significant improvement in dementia clients symptom severity over 18-months • Statistically significant improvement in frequency and length of institutionalisation over 18-months • Statistically significant reduction in utilisation of family services at 18 months	Preliminary level II high quality evidence to support a 6 month dementia family care programme with a needs based intervention with multi-disciplinary input for improving caregiver burden and quality of life and dementia clients symptom severity
Jansen et al., (2011) [25] The Netherlands	*N* = 99 pairs of community-dwelling older adults with dementia symptoms and their primary informal caregivers	• *N* = 54 • Four-months of case management by District Nurses specialised in geriatric care • Case managers coordinated assessments, gave advice and information, monitored care and assisted with planning, organisation and collaboration.	• *N* = 45 • Usual care which included a diversity of health care and welfare services that was accessed depending on people’s own initiative	• Caregivers sense of competence – *Sense of Competence Questionnaire* • Caregivers quality of life *– MOS 36-item Short-Form Health Survey (SF-36)* • Caregivers depressive symptoms – *Center for Epidemiologic Studies Depression Scale (CES-D)* • Burden – *Self-Perceived Pressure by Informal Care* • Patient quality of life – *Dementia Quality of Life Instrument*	• 80 % follow-up data for intervention group, 84 % control group • No differences over time between groups for sense of competency, quality of life, depressive symptoms, burden and patient quality of life	Lack of level II high quality evidence to support 4 months of case management for older adults with dementia symptoms and their primary caregivers to impact on sense of competency, quality of life, depressive symptoms, burden and patient quality of life
Lam et al.*,* 2010 [26] Hong Kong	*N* = 102 Chinese community dwelling people with mild dementia (psychiatric and geriatric patients)	• *N* = 59 • Four-months of Case Management by a trained Occupational Therapist • Regular home visits, assessment and advice, evaluation of the activities of daily living, neuropsychiatric symptoms, caregiver distress and care duties. • Case manager advised caregivers and people with dementia about safe performance in basic self care activities to promote safe home living, behaviour management and communication techniques. Home based program was based on cognitive stimulation • The case manager also worked with the family/person at follow-up hospital clinic visits and liaised with psycho-geriatrician or geriatrician	• *N* = 43 • One home visit for home safely by occupational therapist no case management	Caregivers • *Zarit Burden Scale* • *General Health Questionnaire* • *Personal Well-Being Index for Adult* Persons with Dementia • *Neuropsychiatric Inventory and MMSE* • *Cornell Scale for Depression in Dementia* • *Person-Wellbeing Index for Intellectually Disabled*	• 90 % follow-up data for both groups • None of the changes of primary and secondary outcomes at 4 or 12 months showed significant group differences • At follow-up the case management group used more day care and domestic helpers than the control group	Lack of level II high quality evidence to support a 4 month active case management intervention to reduce caregiver burden in Chinese people with mild dementia in Hong Kong. However there was an increase in external supports in the intervention group.

**Table 4:** Mixed Methods Study Design – Case Manager Roles

Article	Sample	Intervention	Control	Outcome measures	Outcome/results	Conclusion
Iliffe et al.*,* (2014) [27] United Kingdom	*N* = 29 dyads (people with dementia-carer) who were not receiving care coordination from specialist services	• Study aimed to adapt a United States model of primary care-based case management for people with dementia and test it in four general practices: one rural, one inner-city, and two urban practices (CAREDEM study) • The CARDEM intervention consisted of training and mentoring based on an educational needs assessment in conjunction with a learning manual • The trainer and mentor for the case mangers was an experienced Admiral Nurse who visited each workplace and was available by phone and email • The case managers were practice nurses in the rural and inner-city practices and a social worker in the other urban practices	N/A	• Mixed methodology case studies • Quantitative data: numbers identified, eligibility for case management, number and types of needs and number of contacts • In-depth interviews with stakeholders including people with dementia, carers, case managers and their mentor, health and social care professionals and researchers • Case manger records were compared with findings from the interviews	• Sixty-three case manager contacts were recorded and the median number of contacts and type of contacts varied significantly between case managers • The proportion of needs for which actions were recorded varied significantly by type of need for carers but not patients • Researchers identified more unmet needs than case managers • Perceived benefits of case managers identified from carers and people with dementia were: first point of contact, a safety net and creating a one-to-one therapeutic relationship. Some suggested the care managers take a more active role in negotiating with local services • Health care professionals stated the case manager provided continuity of care and was seen as complementary to existing services • Case managers perceived the advantages as the continuity of care and flexibility in responsiveness to needs but wished they had more time to develop their work and show concrete benefits	This mixed methods study showed that case management offered potential benefit to people with dementia, their carers and community based professionals through continuity of care by a named trust individual that could act proactively to prevent a crisis. However, it was also shown that needs may be overlooked. It is suggested that further development work is need to establish the best approaches to meeting the needs of people with dementia and their cares before case management can be implemented in primary care.
Verkade et al.*,* (2010) [28] The Netherlands	*N* = 30 experts in the field of case management (14 practising professionals nine case managers for people with dementia, three team managers, one geriatrician, one psychiatrist)	• *N* = 30 • Modified four-phase Delphi design to build consensus on the essential components that form part of case management programmes for people with dementia and the preconditions needed for effective implementation	N/A	• Literature Review • Focus Group Interview (*N* = 8) • First Delphi survey round to validate the pre-selected items • Second and third Delphi surveys designed to score items with a view to reaching consensus	• Consensus was reached on 61 out of 75 statements. • Essential components were: information, support and counselling, coordination of the care provided, and to a lesser extent practical help. A patient centred approach was found to be one of the key aspects. • Essential preconditions were: vision, care relationship, structured methodology, integration of case management into the health care chain, and the case manager’s level of training/expertise.	It is recommended that the essential components and preconditions be used as a basis for developing minimum quality criteria for case management in people with dementia to enhance quality of care and reduce undesirable differences.

Note: Assessment of bias was not relevant for the mixed method studies as their study design did not meet the criteria for the risk of bias tools; instead the methodology was critiqued according to Greenhalgh & Taylor’s [21] paper and Britten & Pope’s [22]

**Table 5:** Qualitative Study Designs – Case Manager Roles

Article	Sample	Intervention	Control	Outcome measures	Outcomes/results	Conclusion
Minkman et al.*,* 2009 [29] The Netherlands	*N* = 16 Eight regional dementia care provider networks with two respondents from each programme (manager and case manager)	No intervention. Article conducts a multiple case study of case management programs in various regions in the Netherlands to determine their effectiveness. Inclusion criteria included: Case management had to have been implemented for at least 1 year, program documentation such as aims and planning had to be available, and programs had to work with multiple case managers focusing particularly on dementia patients and their caregivers living in the community.	N/A	• Questionnaire (based on a non-systematic literature review for international studies in dementia care). Seven categories: programme history, motives and tasks, patient group and caseload, background and capacities, process, collaboration and implementation success and fail factors. • Semi-structured face-to-face interviews. Guide was developed and reviewed by experts from the National Dementia Programme	• The motives, aims and main characteristics of case management were comparable. • All programmes offered services that focused on increasing the continuity and integration of primary, speciality, mental and long-term health care • Differences in models were in terms of the targeted dementia patient groups as well as the background of the case managers and their position in the local dementia care provider network. • Similarities were identified in terms of vision, tasks, processes and partners. • Factors for success included the expert knowledge of case managers, investment in a strong provider network and coherent conditions for effective inter-organisational cooperation to deliver integrated care.	Future research is recommended on the effects of case management in dementia care that focuses on the individual level of clients and caregivers and the organisation level of the care network. It is also recommended that a cost-effectiveness evaluation be undertaken and outcomes such as caregiver burden, problematic behaviours and well-being and depression be measured.

Note: Assessment of bias was not relevant for the qualitative studies as their study design did not meet the criteria for the risk of bias tools; instead the methodology was critiqued according to Greenhalgh & Taylor’s [21] paper and Britten & Pope’s [22] work

#### Level II evidence - randomised controlled trials

Outcomes of study and effective/non effective components of the model

The four RCTs utilising case management models evaluated the roles impact on outcomes for the carer and person with dementia (Table 2 below).

The two RCTs conducted by Chien and Lee [23, 24] with intervention periods of 6 months produced significant outcomes for people with dementia and carers. These outcomes included: reduction in carer burden and improvement in quality of life]; and reduced institutionalisation rates at 12- [23] and 18-months [24] post intervention. Additionally people with dementia showed improved symptom severity at 6-months [23] and 18-months [24].

The remaining two RCTs with intervention periods of four [26] and 12-months [25] found no significant differences in carer and person with dementia health or social outcomes measured in any of the follow-up assessments (4, 6, 12-months) [25, 26]. However, Lam et al. did show a significant increase in family carers of people with dementia seeking external support at both 4 and 12-months. [26]. Jansen et al. indicated that the lack of significant results may have been attributed to either the intervention being offered too early or it lacking the intensity or duration to achieve a change in outcomes [25].

The interventions showed variance in length and mode of support as well as the role and qualification of the case manager. The components in the case manager roles in the two RCTs producing significant results included: a 6-month intensive intervention; input from a multi-disciplinary committee; training of the case manager; clinical backgrounds (nurses as case managers); collaborative care; continuity of care (same case manager); structured needs assessments and individualised education and support programs for each participant.

### Risk of bias

Overall the methodological quality in three of the four RCTs investigating case manager roles was high [24–26] (see Table 6).

**Table 6:** Risk of bias summary - Details of RCTs included in the study and assessment of the risk of bias of each study according to Cochrane

	Random sequence generation (selection bias)	Allocation concealment (selection bias)	Blinding participants and personnel (performance bias)	Blinding of outcome assessment (detection bias) (patient-reported outcomes)	Incomplete outcome data (attrition bias) (short-term 2–6 weeks)	Incomplete outcome data (attrition bias) (long-term >6 weeks)	Selective reporting (reporting bias)
Case Managers – Randomised Controlled Trials (Cochrane Risk of Bias Tool)							
Chien and Lee 2008 [23] Hong Kong Not registered	?	?	+	+	+	+	?
Chien and Lee 2011 [24] Hong Kong Not registered	+	+	+	+	+	+	?
Jansen et al.*,* 2011 [25] The Netherlands ISRCTN83135728	+	+	+	+	+	+	-
Lam et al.*,* 2010 [26] Hong Kong Not registered	+	+	+	+	+	+	-
Care Managers – Randomised Controlled Trials (Cochrane Risk of Bias Tool)							
Callahan et al.*,* 2006 [47] USA NCT00246896	+	+	+	+	+	+	+
Chodosh et al.*,* 2012 [44] USA ISRCTN72577751	+	+	?	?	+	+	+
Duru et al.*,* 2009 [45] USA ISRCTN72577751	+	+	?	?	+	+	+
Specht et al.*,* 2009 [48] USA Not registered	+	?	-	-	-	-	?
Vickrey et al.*,* 2006 [46] USA ISRCTN72577751	+	+	+	+	+	+	+
Counselling support roles – Randomised Controlled Trials (Cochrane Risk of Bias Tool)							
Bass et al.*,* 2003 [33] USA Not registered	+	?	+	?	?	?	?
Brodaty et al.*,* 2009 [38] UK & USA Not registered	+	?	?	+	+	+	?
Burns et al.*,* 2003 [35] USA NCT00178165	+	?	?	+	?	?	+
Clark et al.*,* 2004 [34] USA Not registered	+	?	+	?	?	-	?
Eisdorfer et al.*,* 2003 [36] USA NCT00178165	+	?	?	?	?	?	+
Fortinsky et al.*,* 2008 [43] USA Not registered	+	+	?	+	+	+	?
Gaugler et al.*,* 2008 [30] USA NCT00362284	+	?	?	?	+	+	+
He’bert et al.*,* 2003 [39] Canada Not registered	+	?	?	+	+	+	?
Mahoney et al.*,* 2003 [37] USA NCT00178165	+	?	?	+	+	+	+
Mittelman et al.*,* 2004 [31] USA NCT00362284	+	?	-	-	+	+	+
Mittelman et al.*,* 2006 [32] USA NCT00362284	+	+	-	?	+	+	+
Nobili et al.*,* 2004 [40] Italy Not registered	+	?	?	?	+	+	?
Teri et al.*,* 2003 [41] USA Not registered	+	+	?	+	+	+	?
Wray et al.*,* 2010 [42] USA	+	?	?	+	?	?	-
Team based/Multi-Agency/Integrated Support Roles – Randomised Controlled Trials (Cochrane Risk of Bias Tool)							
Eloniemi-Sulkava et al.*,* 2009 [49] Finland Not registered	+	+	?	-	-	-	?
Support/Key worker roles – Observational Study Design Analytic Cohort Studies (adapted from the CASP)							
Woods et al.*,* 2003 [52] United Kingdom Not Registered	+	+	?	-	+	-	?
Team Based/Multi-Agency/Integrated Support Roles – Observational Study Design Descriptive Case Report/Case Series (CASP)							
Stevenson et al., 2006 [50] United Kingdom Not Registered	?	-	?	-	-	-	?

- High risk of bias + Low risk of bias ? Unclear risk of bias

#### Mixed methods evidence

Outcomes of study and effective/non effective components of models

Mixed method evaluations of a case management model consisted of quantitative client data and in-depth interviews [27] and a four phase Delphi Survey and focus group [28] (Table 4). Iliffe et al.*,* [27] showed that case management offered potential benefit to people with dementia, their carers and community based professionals through continuity of care by a named trusted individual that could act proactively to prevent a crisis. However, it was also shown that needs may be overlooked. Verkade et al.*,* [28] found that the essential components of dementia case management were: information of the patients and their systems; support to the patients and their systems; coordination and monitoring of the care provided by others and to a lesser extent practical help. It is suggested that the appropriate way of offering case management is through a patient-centred approach and that successful case management requires that case managers be able to rely on a shared case management vision to give direction to day-to-day care provided in practice.

### Risk of bias

The methodological quality of both studies was good in terms of design, recruitment and data analysis. Limitations of the studies included a small sample size [27] and an inability to generalise results to other countries [28].

#### Qualitative evidence

Outcomes of study and effective/non effective components of models

Minkman et al.*,* [29] undertook a qualitative case study analysis in order to describe and analyse a new approach in extensive case management programs concerned with long-term dementia care in the Netherlands (Table 5) [29]. It was found that the success for case management in long-term dementia care concern the expert knowledge of case managers; investment in a strong provider network and coherent conditions for effective inter-organisational cooperation to deliver integrated care. The failure factors were: distrust of the programme by local providers and competition for delivering care; inadequate or no structural funding; little or no involvement of primary care specialists; doubt about the added value of case managers; and not including patients without a confirmed diagnosis of dementia.

#### Risk of bias

The methodology was sound however only one case manager from each program was included in the interviews and no consumers were interviewed to determine their views.

### Counselling support roles

Fourteen RCTs that evaluated counselling support type roles were identified (Tables 7 and 8).

**Table 7:** Counselling Roles RCT Outcomes

Study	Carer outcomes	People with dementia outcomes	Other
Bass et al. 2003 [33]	• Satisfaction with health plan • Depressive symptoms and strain	N/A	• Utilisation (health services)
Brodaty et al., 2009 [38]	N/A	N/A	• Time to nursing home admission or death
Burns et al., 2003 [35]	• Wellbeing • Depressive symptoms • Effect of dementia symptoms on carer bother	N/A	N/A
Clark et al., 2004 [34]	N/A	• Severity of memory problems • Satisfaction with quality of services • Depressive symptoms • Perceived strain due to memory problems (relationship strain, embarrassment, isolation, difficulty coping)	• Utilisation (health services)
Eisdorfer et al., 2003 [36]	• Mental health, wellbeing, depressive symptoms • Burden • Religiosity • Physical health • Medication usage	• Physical health • Medication usage • Behaviour • Cognition	• Utilisation (services)
Fortinsky et al.*,* 2009 [43]	• Self-efficacy • Burden • Depressive symptoms	N/A	• Nursing home admission
Gaugler et al., 2008 [30]	• Burden • Depressive symptoms	N/A	• Nursing home admission
He’bert et al., 2003 [39]	• Frequency and reactions to behavioural problems • Burden • Psychological distress • Anxiety • Perceived social support • Personal efficacy	N/A	N/A
Mahoney et al.*,* 2003 [37]	• Bothersome nature of care giving • Anxiety • Depressive symptoms • Care giving mastery	N/A	N/A
Mittelman et al., 2004 [31]	• Depressive symptoms • Perception of severity of dementia	N/A	N/A
Mittelman et al., 2006 [32]	• Depressive symptoms • Burden • Satisfaction with social support • Physical health	• Functioning • Frequency of memory and behaviour problems • Physical health	• Nursing home placement • Death
Nobili et al., 2004 [40]	• Amount of stress	• Frequency of problem behaviours	N/A
Teri et al., 2003 [41]	• Behavioural disturbance and distress	• Physical health and function • Affective status – depressive symptoms • Behavioural disturbance	N/A
Wray et al., 2010 [42]	N/A	N/A	• Utilisation (health services) • Cost

**Table 8:** Randomised Controlled Trials - Counselling Support Roles – Level II Evidence

Article	Sample	Intervention	Control	Outcome measures	Outcomes/Results	Conclusion
Bass et al., 2003 [33] United States of America Not registered	*N* = 182 primary family caregivers of people with dementia	• *N* = unknown • Added care consultation telephone intervention with on average 12 direct communication contacts per year to managed care services • Care consultants work with families in a collegial fashion to help identify personal strengths, provide information regarding available community services, facilitate decisions about how to utilise and apply these services and may contact service agencies on behalf of participants	• *N* = unknown • Received usual managed care services and could independently contact the Association for services other than care consultation.	• Utilisation outcomes – *number of hospital admissions, number of emergency department visits, number of physician visits* • Caregiver satisfaction with health plan outcomes – indexes of satisfaction • Caregiver Depression and Strain outcomes – similar to CES-D scale	• 86 % follow-up *N* = 157 • No significant intervention effects for utilisation outcomes – intervention group less likely to have case management visits or use direct care community services • Significantly increased caregiver satisfaction with health plan outcomes when the care recipient had not received a specific dementia diagnosis • Caregivers in the intervention group had greater decreases in reported symptoms of depression • Non-spouses caregivers showed decreases in relationship strain over 12-months while there was no effect on spouse caregivers	Preliminary level II high quality evidence for care consultation over a year period to significantly decrease depression symptoms in caregivers and reduced caregiver strain in non-spousal caregivers
Burns et al., [35] United States of America Not registered	*N* = 167 caregiver-care recipient dyads	• *N* = 82 • Enhanced Care: education sessions on behaviour management, 25 pamphlets and 12 additional pamphlets on stress-coping/stress behaviour management • 24-month primary care intervention conducted every 3 months. Behaviour care component but targeted more towards caregiver wellbeing ~ 60 min duration	• *N* = 85 • Behaviour care: education sessions on behaviour management, 25 pamphlets on behaviour modification • 24-month primary care intervention conducted every 3 months ~ 30 min duration	• Caregiver Outcome Data – *General Well-being scale, Center for Epidemiological Studies Depression scale, Revised Memory and Behavior Problems Checklist*	• 46 % follow-up data at 2 years • Significant changes in general well-being over time favouring the enhanced care group • Significant changes in CES-D over time for both groups • Significant decrease in Revised Memory and Behaviour Problems Checklist scores over time for both groups	Preliminary level II high quality evidence for an enhanced care program that focused on managing behavioural problems and assisted with coping strategies to significantly improve general wellbeing in caregivers when compared to a behaviour care education intervention.
Brodaty et al., 2009 [38] Australia, United Kingdom, United States Not registered	*N* = 155 people with Alzheimer’s Disease and their spouses	• *N* = 79 • All participants received donepezil for 24 months • Standard services: resource information, help in an emergency and routine services at each site • Psychosocial intervention: Five counselling sessions within 3 months and ad hoc counselling for up to 2 years	• *N* = 76 • All participants received donepezil for 24 months • Standard services as intervention group but no formal structured counselling	• Caregiver depression – *Beck Depression Inventory* • Social support – *Stokes Social Network List* • Patient assessment – *MMSE, Global Deterioration Scale Alzheimer’s Disease Assessment Scale – cognitive subscale, Alzheimer’s Disease Cooperative Study – Activities of Daily Living and Revised Memory and Behavior Problems Checklist* • Time to nursing home admission or death assed using – *Cox Proportional Hazards model*	• All participant data (*N* = 155) included in analyses • No difference in times to nursing home placement or time to death between groups	Lack of level II high quality for a 2 year counselling intervention to delay nursing home admission or increase survival until death in people with Alzheimer’s Disease
Clark et al., 2004 [34] United States of America Not registered	*N* = 121 people with dementia or an indication of memory loss	• *N* = unknown • Multi-component telephone-based care consultation delivered by one of three staff (two social workers) with on average 10 direct communications per year	• *N* = unknown • Received usual managed care services and could independently contact the Association for services other than care consultation.	• Memory Problems – *Blessed Orientation-Memory-Concentration Test* • Utilisation Outcomes – *Medical record data* • Psychosocial Outcomes – *interviews, Center for Epidemiological Studies Depression Scale, four index item of relationship strain, embarrassment and isolation scales developed specifically for the project*	• 74 % follow-up data *N* = 89 • Intervention group participants had significantly decreased feelings of embarrassment and isolation due to memory problems and decreased difficulty coping with memory problems • Intervention group participants with average or greater than average memory difficulties were significantly less likely to have a hospital admission or emergency department visit during in the 12-month study period and were significantly more satisfied with quality of services	Preliminary level II high quality evidence for a 12-month multi-component telephone-based care consultation intervention to significantly reduce feelings of embarrassment and isolation and decrease ‘difficulty in coping’ due to memory problems in people experiencing memory problems or with a diagnosis of dementia. Additional intervention effects were shown for people with more severe impairment.
Eisdorfer et al., 2003 [36] United States of America Not registered	*N* = 225 caregivers of people with Alzheimer’s Disease	• Resources to Enhance Alzheimer’s Caregiver Health for Telephone-Linked Care (REACH for TLC). 18-months of • Structural Ecosystems Therapy: (structured family therapy intervention for treatment of behaviour problems) *N* = 75 or • Structural Ecosystems Therapy plus Computer-Telephone Integrated System (information network computer-telephone technology to augment the therapeutic intervention by facilitating linkages of caregivers with their family and supportive resources outside of the home) *N* = 77	• *N* = 73 • Minimal Support Control group – bi-weekly phone calls for 6-months and then monthly calls for 12-months (active listening and empathic comments)	• Activities of Daily Living and Instrumental Activities of Daily Living • Caregiver Stress – *Revised Memory and Behavior Problems Checklist, State Anxiety Inventory, Center for Epidemiological Studies Depression scale* • Satisfaction with Social Support • MMSE	• 6-months 65 % follow-up data; 18 months 68 % • Caregivers in the combined family therapy and technology intervention experience a significant reduction in depressive symptoms at 6-months • At 18-months the combined intervention was significantly effective for Cuban American husband and daughter caregivers	Preliminary level II high quality evidence for a combined family therapy and technology intervention in reducing depressive symptoms in caregivers particular in Cuban American husband and daughter caregivers
Fortinsky et al., 2009 [43] United States of America Not registered	*N* = 84 family caregivers of people with dementia	• *N* = 54 • Care Consultation by a Alzheimer’s association chapter with monthly contact for 12-months for family caregivers via telephone (three changes in staff; professions included speech and language specialist and clinical social workers)	• *N* = 30 • Received identical educational materials to intervention group with details on dementia symptom management and available community services no further attention from study personnel	• Nursing Home Admission • Self-efficacy – *symptom management measure and community support service use* • Caregiver burden – *22-item Revised Caregiver Burden Scale* • Caregiver Depression – *Center for Epidemiological Studies Depression inventory* • Caregiver physical health – *Hopkins Symptoms Checklist* • Satisfaction with intervention	• Primary outcome 96 % follow up data, 82 % other dependent variables, 89 % interview data • Family caregivers in the intervention group were less likely to be admitted to a nursing home – however this was not a statistically significant result • There was no statistically significant effect on self efficacy, depressive symptoms, caregiver burden or physical symptoms	Lack of level II high quality evidence for a 12-month care consultation program to significantly lower rates of nursing home admission however there was a trend toward those in the intervention group. There was no significant effect on any secondary outcomes.
Gaugler et al., 2008 [30] United States of America NCT00362284	*N* = 406 spouse-caregivers of people with Alzheimer’s disease who lived at home	• *N* = 203 • Enhanced counselling and support by counsellors with advanced degrees in social work or allied professions (six counselling sessions, weekly support groups and ongoing ad hoc counselling) • 9.5 years of data are reported	• *N* = 203 • Received services provided to all families of patients at the New York University Alzheimer’s Disease Center: no formal counselling	• Nursing Home Admission: interviews • Caregiver Burden – *Zarit Burden Interview* • Caregiver depression – *Geriatric Depression Scale (GDS)*	• 95 % data for primary outcome measure • In both models nursing home admission significantly reduced burden and depressive symptoms • Caregiver burden was significantly lower in the intervention group at each point after nursing home admission • Intervention depression scores were significantly lower than usual care scores at all points before nursing home admission with the exception of baseline. This difference was maintained after nursing home admission for approximately 4 months after which the scores were similar for the remainder of the study	Preliminary level II high quality evidence for nursing home admission reducing caregiver burden and depressive symptoms regardless of the intervention. However six sessions of enhanced counselling and readily available ongoing supportive maintenance provided statistically significant longer term benefits compared to usual care.
He’bert et al., 2003 [39] Canada Not registered	*N* = 144 caregivers of people with dementia	• *N* = 72 • 15 week psycho-educative program focusing on cognitive appraisal and coping strategies by a health professional experienced in the care of people with dementia. • 15 2-hour weekly group sessions	• *N* = 72 • Participants were referred to regular support group program offered by the Alzheimer Society or health care organisations in their region	• Interviews baseline and 16 weeks • Frequency of behavioural and memory problems – *Revised Memory and Behavior Problem Checklist* • Desire to Institutionalise • *Zarit Burden Interview* • Anxiety – *Spielberger State-Trait Anxiety Inventory* • *Bradburn Revised Affect Scale* • *Inventory of Socially Supportive Behaviours* • Personal Efficacy • Psychological distress – *Psychiatric Symptoms Index*	• 82 % follow-up data • There was a statistically significant reduction in disruptive behaviours reaction score in the intervention group • Cross-product frequency/reaction differences between groups was statistically significant	Preliminary level II high quality evidence of a 4 month psycho-educative program to significantly reduce caregiver reactions to behaviour problems
Mahoney et al., 2003 [37] United States of America NCT00178165	*N* = 100 caregivers of people with Alzheimer’s Disease	• *N* = 49 • Resources to Enhance Alzheimer’s Caregiver Health for Telephone-Linked Care (REACH for TLC). • Twelve months of using a computer-mediated telecommunications system. Interactive voice response system rich with Alzheimer’s information, personal mailbox, bulletin board and activity-respite conversation. • Weekly conversation with counsellor	• *N* = 51 • Reference booklet with similar content to module 1 of the intervention that provided strategies to manage AD-related disruptive behaviours	• Activities of Daily Living and Instrumental Activities of Daily Living • Caregiver Mastery Scale • Caregiver Stress – *Revised Memory and Behavior Problems Checklist, State Anxiety Inventory, Center for Epidemiological Studies Depression scale*	• Follow-up: bothersome measure (45 % both groups) depression and anxiety measures (80 % intervention 84 % control) • No overall significant effect on reducing bother scores, depression or state anxiety scores. • Significant decline in bother scores, depressive symptoms and anxious complaints in participants with low-mid mastery at baseline compared to controls • Caregivers who were wives had a significant reduction in the bothersome nature of caregiving compared to controls	Preliminary level II high quality evidence for an automated telecommunications system designed for caregivers of people with Alzheimer’s Disease in reducing bother, depressive symptoms and anxious complaints in caregivers with low mastery and for those who were wives.
Mittelman et al., 2004 [31] United States of America NCT00178165	*N* = 406 spouse-caregivers of people with Alzheimer’s disease who lived at home	• *N* = 203 • Enhanced counselling and support by counsellors with advanced degrees in social work or allied professions (six counselling sessions, weekly support groups and ongoing ad hoc counselling) • Data for the first 5 years is presented	• *N* = 203 • Received services provided to all families of patients at the New York University Alzheimer’s Disease Center: no formal counselling	• Caregiver depression – *Geriatric Depression Scale* • Severity of dementia – *Global Deterioration Scale*	• 80 % follow-up data • At 12-months the change in Geriatric Depression Scale Score was statistically significant • The significantly fewer depressive symptoms in the intervention group were sustained for 3.1 years after baseline	Preliminary level II evidence for a short course of intensive counselling and readily available ongoing supportive maintenance in reducing symptoms of depression among caregivers of people with dementia.
Mittelman et al., 2006 [32] United States of America Not registered	*N* = 406 spouse-caregivers of people with Alzheimer’s disease who lived at home	• *N* = 203 • Enhanced counselling and support by counsellors with advanced degrees in social work or allied professions (six counselling sessions, weekly support groups and ongoing ad hoc counselling) • Data over an 18-year period are reported	• *N* = 203 • Received services provided to all families of patients at the New York University Alzheimer’s Disease Center: no formal counselling	• Dates of permanent nursing home placement and of death were monitored during regular follow-up interviews and telephone contacts. Dates of death confirmed with Social Security Death Index	• All data available for primary endpoint; 97.5 % for interviews • The intervention group had significant delays in nursing home placement – 28.3 % reduction compared to controls	Preliminary level II high quality evidence for a short course of intensive counselling and readily available ongoing supportive maintenance in significantly delaying nursing home placement.
Nobili et al., 2004 [40] Italy Not registered	*N* = 69 people with a diagnosis of dementia and their caregiver	• *N* = 35 • Structured intervention: one home visit by a psychologist and one home visit by an occupational therapist; information manual and list of contacts	• *N* = 34 • Free helpline, information rights and legal aspects, how to file forms for economic help and addresses of community services	• Frequency of problem behaviours – *SBI-C* • Caregiver stress – *RSS* • MMSE • Basic and Instrumental activities of daily living – *ADL and IADL*	• 56 % follow-up data for 12-months • Mean problem behaviour score was significantly lower in the intervention group at 12 months • Significant reduction in frequency of delusions and psychic agitation at 12-months in the intervention group	Preliminary level II high quality evidence for a structured intervention (on two occasions) in reducing frequency of problem behaviour particularly delusion and psychic agitation in people with dementia
Teri et al., 2003 [41] United States of America Not registered	*N* = 153 community dwelling people with Alzheimer’s Disease	• *N* = 76 • Exercise program and behavioural management and education program for caregivers by clinical geropsychologists and a physical therapist • 12 h long sessions over an 11 week period, then three follow up sessions over 3 months	• *N* = 77 • Routine medical care including acute medical or crisis intervention provided at community health care centres	• Physical health and function – *SF-36and Sickness Impact Profile* • Affective status – *Hamilton Depression Rating Scale, Cornell Scale for Depression in Dementia* • Physical Health tests • Patient behavioural disturbance and caregiver distress – *Revised memory and Behavior Problem Checklist*	• 92 % completed post-test assessment; 58 % completed 24-month assessment At 3 months: • Statistically significant improvement in SF-36 and Cornell depression scores At 24 months: • Statistically significant differences between groups on the SF-36 physical role functioning subscale and the SIP Mobility Scale Additional Analysis • People with higher depression scores at baseline improved significantly more at 3-months on the Hamilton Depression Rating Scale and maintained this at 24 months.	Preliminary level II high quality evidence for 6-month exercise training combined with teaching caregivers behavioural management techniques to improve physical health in people with Alzheimer’s Disease
Wray et al., 2010 [42] United States of America NCT00105638	*N* = 158 spousal caregivers of people with dementia	• *N* = 83 • Telehealth Education Program delivered by trained group leaders (social workers and nurse dementia care manger) to groups of up to 8 caregivers for 1 h every 10 weeks	• *N* = 75 • All usual services that Veteran Affairs provides expect for the Telehealth Education Program	• Veteran Health Care Cost and Utilisation Data	• All data included - intention to treat • Significant short-term effect (6-month) on total cost and nursing home cost with a decrease in overall cost of care per patient decreasing in the intervention group compared to the control	Preliminary level II high quality evidence for a 10-week Telehealth Education Program in producing significant short-term decreases in overall and nursing home cost of care for people with dementia

Outcomes of study and effective/non effective components of models

The studies which implemented counselling support type roles focused on a range of outcomes for carer and people with dementia (see Table 7).

Three RCTs [30–32] report on different outcomes from an intervention that provided enhanced counselling and support to carers over a 4 month period. Mittelman and colleagues [31] found that at the 5 year follow up after baseline differences were controlled for cares in the intervention group had significantly fewer depressive symptoms compared to controls. These effects were sustained for 3.1 years after baseline and after nursing home placement or death of the patient. A further report by Mittelman and colleagues [32] found that the intervention group had significant delays in nursing home placement when compared to controls. Gaugler et al.*,* [30] then aimed to determine whether the intervention reduced the burden and depressive symptoms of carers during the transition to nursing home placement. It was found that nursing home placement itself reduced burden and depressive symptoms in carers for both groups but that the intervention resulted in significantly lower burden and depressive symptoms at the time of and after nursing home placement.

Two RCTs [33, 34] reported on different outcomes from the Cleveland Alzheimer’s Managed Care Demonstration. The aim of the demonstration was to evaluate the effect of a 12-month care counselling consultation (a multi-component telephone intervention) delivered within a partnership between a managed health care system and Alzheimer’s Association during the 12-month study period. The intervention was shown to significantly decrease depression symptoms in carers and reduce strain in non-spousal carers [33], significantly reduce feelings of embarrassment and isolation and decrease ‘difficulty in coping’ due to memory problems in people experiencing memory problems or with a diagnosis of dementia [34]. Additional intervention effects were shown for people with more severe impairment. There was less direct impact of the intervention on health service utilisation (hospital, emergency department, physician) with significantly lower utilisation only occurring in services that provided that same types of assistance as the intervention.

A further three RCTs [35–37] were a part of the Resources for Enhancing Alzheimer’s Caregiver Health (REACH) multisite research program. Each RCT implemented a different social and behavioural intervention targeting carers of people with dementia. Mahoney and colleague’s [37] 12 month computer mediated interactive voice response system intervention involving counselling showed no significant effect for the intervention in reducing bother scores, depression or state anxiety scores. However, people who were wives or had low mastery scores at baseline did show a significant decline in bother scores, depressive symptoms and anxious complaints as a result of the intervention. Burns et al.*,* [35] reported on parallel simultaneous interventions (behaviour care versus enhanced care) over a 24-month period and found that carers who received either intervention showed significant improvements for bother associated care recipient behaviours. However, those who received the behaviour care component only, compared with those who also received the stress-coping component (enhanced care), had significantly worse outcomes for general wellbeing and a trend toward increased risk of depression. Eisdorfer and colleague’s [36] combined family therapy and technology intervention which ran for 18-months reduced depressive symptoms in carers at 6-months. However at the 18-month follow-up this result was only sustained for Cuban American husband and daughter carers indicating that the intervention has differing effects according to ethnic group and carer-care recipient relationships [36].

The remaining six RCTs were all independent and involved counselling support roles singularly [38] or combined with: psycho-educative programs [39]; structured education [40]; exercise training and behavioural management techniques [41]; telephone-based education [42]; and care consultation [43]. The counselling roles in all six RCTs were focused on supporting the carer.

One study investigated the effects of a 10-week carer telephone support group intervention on cost of care for the care recipient and found a significant short term cost saving benefit at 6-months compared to usual care [42]. However this result was not maintained at 1 year.

Two of the RCTs with counselling roles implemented for 12-months [43] and 2 years [38] looked at nursing home admission as a primary outcome measure. Brodaty and colleagues [38] found, over an average of 5.4 years, no differences in nursing home placement or mortality between groups. Similarly Fortinsky and colleagues’ [43], intervention did not lead to a statistically significantly lower rate of nursing home admission, although there was a trend in favour of the intervention group during the 12-month study period. Fortinsky and colleagues [43] also found no significant intervention effects on the secondary outcomes of carer self-efficacy, depressive symptoms or burden at 12-months.

The remaining three RCTs showed that: a 4 month psycho-educative program significantly reduced carer reactions to behaviour problems at the 4 month post-test [39], a structured intervention (on two occasions) reduced frequency of problem behaviours particularly delusion and psychic agitation in people with dementia at 12-months [40] and a 6-month exercise training program combined with teaching carers behavioural management techniques improved physical health in people with Alzheimer’s Disease at 2 years [41]. There were no significant effects shown of any of the other outcomes for these three RCTs as listed in Table 7.

The heterogeneity in interventions, variance in outcomes measured and conflicting results meant that the effective and non-effective components of each intervention were unable to be quantified.

### Risk of bias

None of the identified RCTs that analysed counselling support roles met all the criteria for low risk of bias. In all 14 of the identified RCTs evaluating counselling support roles the risk of bias was unclear or high in the majority of categories. Therefore the results cannot be considered as the higher level-two high quality evidence (Table 6).

### Care manager roles

Five RCTs that evaluate interventions trialling the care manager role were identified (Tables 9 and 10). Three of the studies report on different findings from the same RCT [44–46].

**Table 9:** Care Manager Role RCT Outcomes

Study	Carer outcomes	People with dementia outcomes	Other
Callahan et al., 2006 [47]	• General mood (including depressive symptoms) • Health resource use	• Depressive symptoms • Symptom severity • Activities of daily living	N/A
Chodosh et al., 2012 [44]	N/A	N/A	Dementia care quality
Duru et al., 2009 [45]	N/A	N/A	Costs of intervention
Vickrey et al., 2006 [46]	• Service utilisation • Dementia knowledge • Confidence • Mastery of care giving • Health quality of life • Social support • Unmet needs	• Health quality of life	Adherence to dementia guideline recommendations
Specht et al., 2009 [48]	• Health status, wellbeing, stressors, care giving endurance potential	• Cognitive status • Activities of daily living • Kinds and frequency of behaviours	N/A

**Table 10:** Randomised Controlled Trials - Care Manager Roles – Level II Evidence

Article	Sample	Intervention	Control	Outcome measures	Outcomes/results	Conclusion
Callahan et al., 2006 [47] United States of America NCT00246896	*N* = 153 older adults with Alzheimer’s Disease and their caregivers from two large primary care practices. Physicians were randomised not participants.	• *N* = 84 • One year of care management and education for the caregiver by an interdisciplinary team lead by an advanced practice nurse integrated within primary care • Standard protocols were used to initiate treatment and identify, monitor and treat behavioural and psychological symptoms of dementia, stressing non-pharmacological management. • Intervention participants all recommended for cholinesterase inhibitors	• *N* = 69 • Augmented usual care including counselling, written educational material and referral to community resources	Interviews at 6, 12 and 18 months with: • Neuropsychiatric Inventory (NPI) • Activities of Daily Living • Health care resource use • Telephone Interview for Cognitive Status Caregivers: • Cornell Scale for Depression in Dementia for the patient • Caregiver portion of NPI • Patient Health Questionnaire-9 • Alzheimer’s Disease Cooperative Study health resource use questionnaire	• No loss to follow-up • Intervention group significantly fewer behaviour and psychological symptoms of dementia measured by the total NPI scores at 12 and 18 months • Caregivers had significant improvements in stress (caregiver NPI) at 12 months but not 18 months • Significant improvement in caregiver depression at 18 months (patient health questionnaire scores) • No group differences in CSDD < cognition, activities of daily living, rates of hospitalisation, nursing home placement or death.	Preliminary level II high quality evidence for 1 year of collaborative care management for people with Alzheimer’s Disease and their caregivers in significantly reducing behavioural and psychological symptoms of dementia and stress and depression in carers when compared to augmented usual care.
Chodosh et al., 2012 [44] United States of America ISRCTN72577751	*N* = 408 older adults with dementia and their caregivers from 18 primary care clinics Secondary analysis of intervention arm data (*N* = 238) from Vickrey study (2006) [46]	• *N* = 238 • More than 12 months of a disease management program led by trained dementia care managers (primarily social workers)) in a health care organisation and community agency • An Internet based care management software system was used for care planning and coordination • Care manager collaborated with the caregiver to: prioritise problem areas; teach problem-solving skills; initiate care plan actions; and send an assessment summary, a problem list, and selected recommendations to the patient’s primary care physician and other designated providers. • The care management protocol included ongoing follow-up, usually by telephone, with frequency based on need and a formal in home reassessment every 6 months to assess the need for major care-plan revisions. • Each dyad could have one or more community agency care managers	• No control group in this analysis. • Encounters with healthcare organisation care managers, community agency care managers and healthcare organisation primary care providers over 18-months were compared.	• Encounters with healthcare organisation care managers, community agency care mangers and healthcare organisation primary care provides over 18-months • Quality domains of assessment treatment, education. support and safety measured from medical records and caregiver surveys	• Exposure to any care management provider type resulted in significantly higher mean percentages of met dementia quality indicators across all four domains • The successive addition of case management exposure types demonstrated a significant increase in the mean percentage of indicators met within all four domains • Statistically significant association between higher levels of met indicators in all four quality domains and increasing frequency of healthcare organisation care managers encounters per month	Preliminary level III-2, evidence for healthcare organisation care managers to improve quality of dementia care over a 1 year period in a case managed intervention group. Additional coordinated interactions with primary care and community agency staff yielded even higher quality of care.
Duru et al.*,* 2009 [45] United States of America	*N* = 408 older adults with dementia and their caregivers from 18 primary care clinics Cost evaluation analysis of Vickrey study (2006) [46]	• *N* = 238 • Intervention same as Chodosh et al.*,* 2012 [44]	• *N* = 170 • Usual care – not offered any of the intervention protocols	Caregiver surveys at baseline, 12 months and 18 months to collect information on: • Patient healthcare utilisation • Paid and unpaid care giving hours • Costs of paid nonprofessional caregivers • Out of pocket expenses	• 71 % follow-up data for intervention group, 74 % control group • No significant differences in inpatient or outpatient utilisation or mean monthly cost of healthcare and care giving services	Lack of level II, high quality evidence for a 1 year dementia care management intervention to lower costs or provide a significant cost offset compared to the costs of usual care at 18-month follow up.
Specht et al., 2009 [48] United States of America Not registered	*N* = 8 countries enrolled 249 client dyads with a minimal inclusion criteria of memory impairment	• *N* = 167 • Dementia Nurse Care Manager provided a model of dementia care for people with dementia and their caregivers. At least monthly contact with continually availability by phone.	• *N* = 82 • Traditional case management service. Monthly phone contact and quarterly face-to-face contact emphasise on coordination of services not delivery of direct services	• Care recipient outcomes – *MMSE, Global Deterioration Scale, Lawton and Brody’s modified IADL/ADL measure, Behaviour Rating Checklist* Caregiver Outcomes – *health status, well-being, stressors, endurance potential, MOS-36 SF, Nursing Outcomes Classification*	• 64 % follow up data intervention; 49 % control • No significant differences in care recipient outcomes between groups • Caregiver outcomes stress, well-being and endurance potential significantly improved in the intervention group and this improvement was consistent over time.	Preliminary level II high quality evidence for a Dementia Nurse Care Manager intervention to significantly improve caregiver stress, well-being and endurance potential over time when compared to a traditional case management service.
Vickrey et al., 2006 [46] United States of America	*N* = 408 older adults with dementia and their caregivers	• *N* = 238 • Intervention same as Chodosh et al.*,* 2012 [44]	• *N* = 170 • Usual care – not offered any of the intervention protocols	Adherence to 23 dementia guideline recommendations at follow-up (four domains: assessment, treatment, education and support and safety) obtained by: • Medical records • Caregiver surveys Secondary outcomes: • Caregiver surveys measuring a range of quality of life and health outcomes	• 12-month response rate 88 %, 18-month 82 %, Medicare data 97.5 % • Mean percentage of per-patient guideline recommendations to which care was adherent was significantly higher in the intervention group • Participants who received the intervention had significantly higher care quality on 21 of 23 guidelines	Preliminary level II, high quality evidence for a 1 year dementia-guideline disease management program to improve quality of care for people with dementia

#### Level II evidence - randomised controlled trials

Outcomes of study and effective/non effective components of models

Four of the five study’s care manager interventions ran for a 12-month period and were specifically designed for people with dementia and their carers in a primary practice setting [44–47]. The further RCT care management intervention was implemented for 15-months and targeted people with dementia and carers already integrated within traditional case management systems in eight different countries [48]. Outcomes measured are shown below in Table 9.

The three RCTs reporting on the same care manager intervention revealed higher quality of care in regard to dementia guideline recommendations [46], that healthcare organisation care managers were essential for dementia care quality improvement and that additional coordinated interactions with primary care and community agency staff yielded even higher quality of care for people with dementia [44]. However, no significant cost offsets for the intervention were found [45].

The remaining two care manager RCTs produced some significant health outcomes, mainly for carers during the intervention period. This included: significant improvements in stress at 12-months (but not 18-months) [47] and significantly improved carer stress, well-being and endurance potential at 15-months [48]. Callahan and colleagues [47] also showed their trial of care management to significantly reduce behavioural and psychological symptoms of dementia during and post-intervention (18-months). However neither intervention showed significant improvements in depressive symptoms, activities of daily living, cognitive status or kind and frequencies of behaviours for people with dementia during or post-intervention [47, 48].

Components of care manager roles that were successful and consistent across interventions included: a 12-month intervention; collaborative care planning; education for people with dementia/their carers; structured assessments; and multi-disciplinary input/collaboration.

#### Risk of bias

The RCTs undertaken by Callahan et al., [47] and Vickrey et al., [46] showed low risk of bias for all assessment indicators indicating high methodological quality (Table 6). Chodosh et al., [44], Duru et al., [45] and Vickrey et al., [46] did not state in any of their three papers whether there was any blinding of personnel or outcome measures. The intervention by Specht et al., [48] had the lowest methodological quality with only category being rated as low risk of bias (random selection generation, selection bias) (Table 6).

### Team-based/Multi-agency/Integrated support roles

The three studies whose interventions involved team-based roles including a RCT [49], observational case report [50] and one qualitative study [51] (Tables 11, 12 and 13).

**Table 11:** Randomised Controlled Trials - Team based/Multi-Agency/Integrated Support Roles – Level II Evidence

Article	Sample	Intervention	Control	Outcome measures	Outcomes/results	Conclusion
Eloniemi-Sulkava et al.*,* 2009 [49] Finland	*N* = 125 community-dwelling couples with one spouse caring for the other spouse with dementia	• *N* = 63 • Maximum 24-month multi-component intervention with a family care coordinator (trained public health registered nurse with dementia education), a geriatrician, support groups for caregivers and individualised services	• *N* = 62 • Continued in usual community care and received care and services from the municipal social and healthcare system, the private sector or both depending on their own initiative.	• Primary Outcome – time from enrolment to long-term institutionalisation • Functional and Wellbeing measures – *Barthel Index, Neuropsychiatric Inventory, Zarit Burden Scale*	• 100 % data for institutionalisation and deaths. Intention to treat used. • At 1.6 years statistically more people with dementia had been admitted to long-term institutional care however at 2 years this was no longer statistically significant • Significant decrease in costs of municipal social and healthcare services in the intervention group compared to the control however when the intervention costs are included this result is no longer significant	Lack of level II, high quality evidence for a 24-month multi-component support program including a family care coordinator, a geriatrician, goal-orientated peer support groups and individualised services to significantly delay long term-institutionalisation of people with dementia.

**Table 12:** Observational Study Designs: Descriptive Studies (case report/case series) – Team-Based/Multi-Agency/Integrated Support Role –Level IV Evidence

Article	Sample	Intervention	Control	Outcome measures	Outcomes/results	Conclusion
Stevenson et al., 2006 [50] United Kingdom	*N* = 65 people with dementia *N* = 28 health workers, social service workers and voluntary sector organisations (surveys) *N* = 8 carers (semi-structured interviews)	• *N* = 22 people with dementia experienced a multiagency enhanced community assessment and support team (EAST) with three care workers and two team coordinators (social worker and a registered mental health nurse) based in the local health centre. Coordinated and comprehensive assessment and care management service was provided to older people with a confirmed diagnosis of dementia • *N* = 23 people with dementia referred to a psychogeriatric admission ward • *N* = 20 people with dementia referred to a psychogeriatric day hospital	No control group	• Naturalistic, descriptive, survey for a 1 year period • Assessment of needs (CarenapD) • Activities of daily living: Bayer-Activities of daily living (BAYER ADL) • Mini-mental state examination (MMSE) • Behavioural psychopathology in Alzheimer’s Disease (BEHAVE-AD) • Surveys with health workers, social service workers and voluntary sector organisations	• 64 % of surveys returned. 78 % found EAST beneficial in management of the referred individual and 94 % agreed that it was useful. • Carers found EAST beneficial and appreciated the regularity of visits, the monitoring, attention and emotional support, the practical assistance, advice and information and the improved awareness and access to resources. • Number of unmet needs in EAST group was initially 13 and reduced to seven • 9 % of people initially referred to EAST required psychogeriatric inpatient assessment and none required day hospital support • 68 % of EAST participants were maintained in their own homes • Use of psychogeriatric day hospital placements and inpatient assessment beds reduced; no EAST participants required admission to the psychogeriatric day hospital.	Preliminary level IV evidence for a multiagency community team EAST to comprehensively assess and support at home patients with dementia who previously would have been referred to the local psycho geriatric admission ward and day hospital, with a consequent reduction in the utilisation of these hospital facilities. Health workers, voluntary agencies and carers were positive about the service.

**Table 13:** Qualitative Study Designs – Team based/Multi-Agency/Integrated Support Roles

Article	Sample	Intervention	Control	Outcome measures	Outcomes/results	Conclusion
Rothera et al.*,* (2008) [51] United Kingdom	*N* = 82 • 27 service users (people aged 65 with diagnosis of dementia or known to the service as having cognitive impairment) • 18 family carers • 17 home care workers • 20 health/social care professionals, across both services	• Specialist multi-agency home care service for older people with dementia introduced in two areas of Nottingham in 1999. • Aimed to reduce high levels of care home placement and respond to statutory inspection recommendations	N/A	• In-depth semi-structured interviews (older people with dementia, family carers, care workers, health professionals and social services managers) • Focus groups (with family carers and care workers) and • Small group interviews (with older people with dementia)	• Five overall categories emerged which summarised the major differences between the services, encompassed the views of all groups and provided a rationale for why the specialist service was better than the standard service. These categories were structure and function; responsiveness; control and autonomy; building relationships; and reducing carer burden. • The specialist service demonstrated greater flexibility and responsiveness to the particular needs and circumstances of service users and family carers, who were encouraged to take part in routine decision making and activities. • By sharing responsibilities, the specialist service helped reduce carer stress and prevent crises. • These outcomes depended on the configuration of the service, including multidisciplinary health and social services input, care worker autonomy and independence, continuous reassessment of clients’ circumstances and preferences and the capacity to develop long term relationships, through care worker continuity. The standard service, which used a task oriented approach, lacked these characteristics.	This qualitative study provides evidence of the benefits of a specialist multi-agency home support service over standard home care in the opinions of service users, carers and care workers.

Note: Assessment of bias was not relevant for the qualitative studies as their study design did not meet the criteria for the risk of bias tools; instead the methodology was critiqued according to Greenhalgh & Taylor’s [21] paper and Britten & Pope’s [22] work

#### Level II evidence - randomised controlled trials

Outcomes of study and effective/non effective components of models

Eloniemi-Sulkava and colleagues*,* [49] aimed to determine whether community care of people with dementia could be prolonged with a 2-year multi-component intervention program that included a family care coordinator, a geriatrician, support groups for care givers, and individualised services [49]. The effects of the intervention on total usage and expenses of social and healthcare services were also analysed. At 1.6 years, a larger proportion of people in the control group were in long-term institutional care when compared to the intervention group however, at 2 years, the difference was no longer statistically significant. The intervention did lead to a reduction in use of community services and expenditure however when the intervention costs were included this result was no longer significant.

#### Risk of bias

There was a high risk of bias with the majority of bias categories being rated as high or uncertain (Table 6).

#### Level IV evidence - observational descriptive (case series/case report)

Outcomes of study and effective/non effective components of models

Stevenson et al., [50] used a naturalistic study design to evaluate the impact of a multi-agency enhanced community assessment and support team that provided coordinated care management services to older people with a confirmed diagnosis of dementia [50]. The multiagency community team comprehensively assessed and supported at home patients with dementia who previously would have been referred to the local psycho geriatric admission ward and day hospital, and consequently reduced utilisation of these hospital facilities. Health workers, voluntary agencies and carers were positive about the service.

#### Risk of bias

Overall the study methodology was poor with no clear information provided on how the data was collected or analysed (Table 6). In addition, no comparative statistical analysis was performed and there was no randomisation of participants meaning that any changes observed cannot be solely attributed to this model of care under evaluation.

### Qualitative evidence

Outcomes of study and effective/non effective components of models

Rothera et al.*,* [51] used a qualitative approach and soft systems methodology to compare a specialist multi-agency home care service for older people with dementia to a standard service in a demographically similar area. The specialist multi-agency home support service demonstrated greater flexibility and responsiveness to the particular needs and circumstances of service users and family carers, who were encouraged to participate in routine decision-making and activities. By sharing responsibilities, the specialist service helped reduce carer stress and prevent crises. These outcomes depended on the configuration of the service, including multidisciplinary health and social services input, care worker autonomy and independence, continuous reassessment of clients’ circumstances and preferences and the capacity to develop long-term relationships, through care worker continuity. The standard service, which used a task-orientated approach, lacked these characteristics.

#### Risk of bias

The methodology used was sound but given the design the results are susceptible to researcher bias, acquiescence bias, inconsistency in the identification of outcomes and false attribution of causation.

### Key worker/Link worker/Admiral Nurse/Clinical nurse consultant roles

The seven studies whose interventions involved support worker roles included one observational analytic cohort and six qualitative studies (Tables 14 and 15). Four of the seven studies evaluated the Admiral Nurse role [52–55] and the remaining three job roles included key workers [56], link workers [57] and a clinical nurse consultant [58].

**Table 14:** Observational Study Design: Analytics Studies (cohort studies) - Support/Key Worker Roles – Level II – 2 Evidence

Article	Sample	Intervention	Control	Outcome measures	Outcomes/results	Conclusion
Woods et al.*,* (2003) [52] United Kingdom	*N* = 128 carers of people with dementia who were new referrals to participating services (admiral nurse services or conventional services)	*N* = 55 Admiral Nurse service with experience mental health nurses with special interest and additional training in dementia care Focus primarily on carers, work exclusively where there has been a diagnosis of dementia, and may continue to provide support after the person with dementia has entered residential care or has died	*N* = 73 Conventional services: multi-disciplinary community mental health teams (occupational therapists, clinical psychologists, psychiatrists, social workers) See work with the caregiver as secondary to the person with dementia Usually do focus on dementia and support is no longer provided if client moves into residential care or if the person dies.	Caregiver strain and distress: *General Health Questionnaire* Institutional placement Severity of Dementia: *Clinical Dementia Rating Scale* *Quality of Relationship*	81 % follow-up data; 104 interviews at follow-up (43 Admiral Nurse, 61 comparison) Significant between group differences for sub-scales anxiety and insomnia favouring the Admiral Nurse group on the General Health Questionnaire No significant differences in outcome for the person with dementia in relation to survival at home Significant reductions in General Health Questionnaire scores for both groups Quality of the pre-morbid relationship between carer and the person with dementia was associated with distress at follow-up	Preliminary level IV evidence for both a conventional multi-disciplinary community mental health service and Admiral Nurse service to result in lower distress scores for caregivers over an 8-month period. Caregivers receiving the Admiral Nurse service also showed a greater reduction in anxiety and insomnia that those receiving a conventional service. Outcomes for people with dementia (in terms of institutional placement) were no worse in the Admiral Nurse group, despite the carer focus.

**Table 15:** Qualitative Study Design – Support Worker Roles

Article	Sample	Intervention	Control	Outcome Measures	Outcomes/Results	Conclusion
Boughtwood et al., (2011) [57] Australia	• *N* = 24 multicultural community link workers from four Australian culturally and linguistically diverse communities (Arabic, Chinese, Italian and Spanish)	• Focus of this study was on workers’ perspectives on the dynamics and management of family caregiving for dementia in culturally and linguistically diverse communities • Multicultural workers provide health education and promotion, community development, information and support groups, and to a more limited extent case work	N/A	• Interviews with multicultural workers • Field notes with description of participants, settings, reflections on interview	• Three main themes were identified: cultural and familial norms pertaining to illness and older people; understanding and naming the term ‘carer’; and patterns in family caregiving. • A number of sub themes were also identified including: keeping dementia in the family; judged by the community; women as carers; children carers; spousal carers; and family sharing care. • (e.g. expectation that elderly people would be cared for by one or more family members usually women, variations of “keeping dementia in the family”	This qualitative study found that multicultural workers perceive and experience many different influences on decisions made about family caregiving including: cultural expectations about what is seen as appropriate behaviour for individuals and families as well as the relationship carers have with the person living with dementia which was sometimes perceived as linked to culture and practical considerations like financial commitments.
Burton et al., (2005) [53] UK	• *N* = 16 client cases • *N* = 2 Admiral Nurses interviewed 16 times about the individual cases	• The aim of the study was to examine the individual decision-making processes of Admiral Nurses in relation to referral management including: how decisions were made regarding referrals and what factors influence this decision making process	N/A	• Case file analysis of cases to identify appropriate cases over a 6 month period for detailed exploration • Interviews with Admiral Nurses	• Four themes influence Admiral Nurse’s decision making: Complexity of carer’s situation; Admiral Nurses’ perception of their specialist role; mode of referral and information received and cross-functional working/trust-wide provision. • The most significant factor that came out of the data was the perceived complexity of the presenting situation, one in which both the circumstances of the carer and the person with dementia were considered. It was also shown that decision-making was influenced where significant risk was identified to either party.	This qualitative study found that the decision to offer the Admiral Nursing service to carers was influenced not only by perceived need but also by the nurses feeling professionally responsible for perceived gaps in service provision. It is suggested that Admiral nurses may need to limit their involvement with carers in line with the service aspirations and become more confident in promoting on-referrals and discharging individuals from the service. It is concluded that it does not appear practical for Admiral nurses to provide a specialist service that meets the needs of all the carers who require support.
Dewing et al., (2005) [54] UK	• *N* = 11 Admiral Nursing teams within England, with two or three Admiral Nurses in each team. The teams were based within existing National Health Service or another provider organisation	• The aim of the study was to work collaboratively with Admiral Nurses to facilitate the development of a competency framework that reflects the needs of the Admiral Nursing Service; to provide a way to structure evidence demonstrating evolving competency and to specifically enable the nurses to demonstrate evidence of achieving the UK Nursing and Midwifery Council’s Higher Level Practice standard.	N/A	• Emancipatory action research and systematic practice development • Observations and in-depth interviews with stakeholders (Admiral Nurses, service managers, and staff and trustees from among the project commissioners)	• Main outcome was development of a specialist nursing competency framework. • The competency framework was made up of a set of eight core competencies: therapeutic work/interventions; sharing info about dementia and carer issues; advanced assessment skills; prioritising work load; preventative and health promotion; ethical and person centred care; balancing the needs of the carer and the person with dementia; promoting best practice. • There were also process-derived outcomes associated with combining systematic practice development with an emancipatory action research design that had an impact on the culture • The main outcomes were that practitioners engaged in and experienced learning about how to research their own practice and the consequences of doing this which are mainly research method findings. • There was some increase in awareness about the culture within the teams and organisations.	This qualitative study developed a competency framework that reflected the needs of the service, was owned by the majority of practitioners and project commissioners which had a positive impact on implementation. It is suggested that the competency framework will enable Admiral Nurses to demonstrate their level of specialist practice as individuals and as a service while also promoting the principles of nurses as lifelong learners.
Duane et al., (2013) [58] Australia	• *N* = 9 people aged over 65 years, with a 6 month history of cognitive decline and functional decline but who had no previous diagnosis of dementia and cognitive impairment in the absence of delirium were included in the study. • *N* = 11 health professionals (focus groups)	• Participatory action research used to refine the role of a Clinical Nurse Consultant specialist in Dementia. • Clinical Nurse Consultant specialist in Dementia role included provision of pre-diagnosis support to people with dementia and their carers/families.	N/A	• Field notes (reflective practice methods) • Semi-structured interviews with participants and their cares • Focus groups with home care nursing service staff and aged care assessment service staff	• The role of a clinical nurse consultant dementia was highly regarded by clients and other health professionals. • It was successful in providing timely assistance and support for consumers and support for other health professionals. • Important aspects of the role included assistance with adjusting to changes in cognition, the relational aspect of the CNC role and opportunities for people with dementia and their carer’s and families to explain their needs and concerns in a time and manner of their preference.	This qualitative study suggests that an inclusive model of community nurse care led by a specialist dementia Clinical Nurse Consultant was successful in providing timely assistance and support for consumers and support for other health professionals. Further research into service provision and evaluation are recommended.
McGhee et al., (2010) [56] Glasgow, UK	*N* = 36 • 18 key workers • 18 carers • Carers were identified solely by the patient’s consultant psychiatrist (purposive sample). Key workers identified by the carer.	• Aim was to create a theoretical explanation of the development of the relationship between key workers and lay carers involved in the care of an individual with dementia	N/A	• Semi-structured, iterative interview to explore participants’ views of the carer-key worker relationship	• A theoretical explanation for the carer/key worker relationship as a complex reciprocal process was described. • Results describe how the relationship may be initiated, strengthened (e.g. through validating and appreciating the carer’s work effort and boosting the confidence of the carer) and managed, but also how it can be weakened (e.g. if the carer adopts a position/view that they are the only individual to be involved in providing care or alternatively the key worker pushes a very dominant approach) and other mediating influences on the relationship. • Very little information is provided in this paper as to the key worker role itself. • Overarching theory of the reciprocal relationship is illustrated in a diagram.	This qualitative study has produced a model that provides a framework for further research into the psychosocial aspects of care giving. The theory requires further empirical study to allow for a more confident prediction that these propositions will produce the benefits for this relationship. There are implications for health care professionals working within the field of dementia care as well as those providing care/support to a close relative or friend living with dementia.
Quinn et al., (2013) [55] UK	• *N* = 6 dyads (six female spousal caregivers and six male care recipients) • *N* = 3 Admiral Nurses	• Study of relationship between Admiral Nurses (ANs), caregivers and care recipients. The aim was to explore how the members work together with this triadic context.	N/A	• Semi-structured interviews with dyads and Admiral Nurses • Case studies were then created presenting perspectives of the caregiver, the care-recipient and the Admiral Nurse	• The case studies were encompassed under an overarching process the authors call “negotiating the balance”, i.e. the ongoing struggle of the members to balance the views of other members against their own needs emerged. The process is seen as dynamic as it is constantly changing. • There was evidence of coalitions occurring between the caregivers and the Admiral Nurses and between the caregivers and care-recipients. It is also showed that coalitions can also arise between the Admiral Nurses and the care-recipients. • There was evidence of both enabling and disabling dementia communication where care-recipients were encouraged to express their feelings and participate in decision-making but where also, in some cases, discouraged from expressing their thoughts and excluded from decisions. Though negotiation was present. • Admiral Nurses perceived that some care givers had difficulty attributing care recipients changes in personality and behaviour to dementia.	This qualitative study showed that the differences in the views of the triad influenced the way they worked together and negotiating the balance of the interactions influenced the effectiveness of the support provided by the Admiral Nurses. It is suggested that longitudinal studies are need to explore how the relationship between the triad changes over time as the negotiations continue to try and reach a balance.

Note: Assessment of bias was not relevant for the qualitative studies as their study design did not meet the criteria for the risk of bias tools; instead the methodology was critiqued according to Greenhalgh & Taylor’s [21] paper and Britten & Pope’s [22] work

#### Level IV - observational study designs: analytic studies (Cohort Studies)

Outcomes of study and effective/non effective components of models

Woods et al.*,* [52] aimed to provide an evaluation of the outcomes association with the Admiral Nurse Service for both the family carer and the person with dementia in comparison to conventional multi-disciplinary community mental health teams for older people in similar areas [52]. Both services resulted in lower distress scores for carers of people with dementia over an 8-month period however carers receiving the specialist Admiral Nurse Service showed a greater reduction in anxiety and insomnia. Outcomes for people with dementia (in terms of institutional placement) were no worse in the Admiral Nurse group, despite the carer focus.

#### Risk of bias

The methodological quality of the trial was good, however given the trial was not randomised and therefore unidentified differences between interventions would have existed and may have influenced the findings.

#### Qualitative

Outcomes of study and effective/non effective components of models

#### Admiral nurse role

Three studies undertook qualitative analyses of the Admiral Nurse role [53–55]. The studies focused on different aspects of the role and service and therefore it was not possible to synthesise their findings. Data collection techniques included structured interviews [53]; emancipatory action research [54] and semi-structured interviews [55]. The studies showed that:

- The desire of Admiral Nurses to fulfil a case management role while attempting to provide a service that is of a specialist nature and of limited capacity generated tension in the role. It was determined that it is not practical for Admiral Nurses in the UK to provide a specialist service that would meet the needs of all those carers who require support and that in order to maximise potential there is a need to further define the services’ remit and enhance its level of specialism [53]
- The development of a specialist nursing competency framework for the Admiral Nurse role in the UK to demonstrate the level of Admiral Nurses specialist practice and core competencies of the role. These were: therapeutic work (interventions); sharing information about dementia and carer issues; advanced assessment skills; prioritising work load; preventative and health promotion; ethical and person centred care; balancing the needs of the carer and the person with dementia; and promoting best practice [54]
- The triadic relationship between the carer, care-recipient and the Admiral Nurse was encompassed under ‘negotiating the balance’ as an overarching process. The findings emphasised the importance of exploring the perspective of all three members in order to improve the quality of support that is provided [55]

The remaining three qualitative studies evaluated three different support worker roles: a key worker [56], link worker [57] and clinical nurse consultant [58]. Data collection methods included grounded theory [56], an empirical investigation [57] and participatory action research [58]. There was qualitative evidence for:

- Positive outcomes in the carer/key worker relationship to be linked to the quality of the relationship and involve the carer and professional care worker actively including and working with the person with dementia [56]
- Link workers to perceive and experience many different influences on decisions made about family caregiving. A shared approach to care was found to be vital in decreasing burden among family members and that due to their close relationship and knowledge of families, multicultural workers can offer an important perspective that is invaluable in informing the provision of carer education and support within CALD communities [57]
- A dementia Clinical Nurse Consultant to show benefit to those living with cognitive impairment and or/their carers and families. The importance of the relational aspect of the role including face-to-face contact and opportunities to explain their needs and concerns in a time and manner of their preference were found to be integral to the person with dementia and carer’s ability to adjust to change [58]

#### Risk of bias

The six qualitative studies discussed above all had limitations with their methodological design. Some of this was due to a lack of clarity around how the data was collected and analysed and some was related to more serious issues such as researcher bias, recruitment bias, limited data analysis methods, low sample sizes or the utilisation of the wrong methodological approach. An overall limitation of these qualitative studies is that the results are quite specific to the population and setting under investigation and thus cannot be generalised to other settings or communities.

## Discussion

Our systematic review of the international literature on models of support for people with dementia and their carers revealed 36 papers which were evaluated in this review.

Systematic reviews of dementia support worker roles have been undertaken previously. These reviews have primarily focused on case management roles and not any of the other support models of care identified in this review. These reviews have investigated case management’s impact on: health care costs and resource utilisation [8]; general wellbeing [9]; consumer and client outcomes [10]; risk of long-term care placement [11]*;* clinical outcomes and utilisation of resources [12] and its potential for people with dementia [13] and barriers to implementation [14]. One systematic review by Low et al. [15] looked at outcomes for older adults including those with dementia from three different models of care: case management, integrated care and consumer-directed care. This review builds on previous reviews, as it is the first of its kind to analyse the essential components of multiple key worker type support roles for people with dementia. The results of this review have the potential to inform future research and practice through the incorporation of these essential features into future trials or current support worker roles operating in the community. The results from our comprehensive systematic review of support models for people with dementia and their carers provide level 1 evidence in regard to evaluations of current models of support for community-dwelling people with dementia and their carers nationally and internationally.

The findings from the systematic review that positively changed characteristics of programs compared to those that did not lead to change suggest that the essential components for support worker roles/interventions were:

- Having an intervention duration of at least 6–12 months in order to significantly impact on measures such as carer burden, general health or wellbeing measures or the person with dementia’s symptom severity
- Having a multi-disciplinary/inter-disciplinary team
- Having collaborative input to determine what support is needed/provided (e.g. with the person with dementia, their carer and family)
- Inter-professional collaborations and a shared approach to care
- Providing individualised support for each person based on a needs assessment
- Ensuring the support worker has a skilled background (e.g. a nurse, occupational therapist, social worker, trained in dementia)
- Providing ongoing follow-up (home visits, telephone contact) that is based on needs
- Providing individualised education based on needs
- Investment in a strong provider network including linking with and having close contact with the physicians/GPs of the person with dementia and coordination and monitoring of care
- Capacity to develop relationships

While the models we examined were categorised according to the definition of the type of support worker there were similarities in the support provided by the workers. It could be inferred that the chosen terminology was just used by the authors as a way to define various multi-component interventions that were under investigation. In fact on closer inspection, many of the models identified: case management/support workers/key workers/link workers/Admiral Nurses were performing very similar roles e.g. information provision and education, referrals to services, and support and advice yet none of the roles were uniform across the studies. Many of the studies identified and previous systematic reviews have only reviewed roles classified as case management. Case management has been defined as “a process encompassing a culmination of consecutive collaborative phases that assist clients to access available and relevant resources necessary to the client to attain their goals” [59]. Taking this definition into account all of the roles identified above in some way performed according to this definition. It is therefore important for future research to further concentrate on determining which aspects, of all support worker type roles, provide the most benefit for people with dementia, their carers/families so that these key features can be incorporated into roles being implemented in practice.

The inconsistencies in results between the studies identified in this systematic review were notable. The heterogeneity in inclusion criteria, design, study populations, recruitment strategies, methods of delivery, role implemented, outcomes measured and the health and social care systems in which they are conducted made it difficult to synthesis results and draw conclusions. It should also be noted that the methodological quality of the majority of the studies included in this review was quite low. Only four studies were rated as having high quality according to the quality criteria. The majority of the trials lacked blinding and allocation concealment (or didn’t clearly state their method) which compromised their quality. Some studies were also underpowered to detect statistically significant differences in effect size between the intervention and control groups. Very few of the RCTs were registered with a clinical trials register and therefore it was not possible to determine if selective reporting occurred. Many of the studies did not describe their data analysis techniques in enough detail which limited the validity and reliability of their results. Mention of confounding factors and the methods used to control for these confounders was also low.

### Implications for research

It is vital that any future research in this area has sound methodology and that the interventions and trials are rigorous in design and delivery. The outcome measures need to be valid and reliable and the methodology clearly defined and well-documented to enable critical appraisal and interpretation of results. With clearly defined sound-methodology there is less risk that the results and outcomes will be subject to bias. In addition to quantitative outcome measures, a qualitative component included in the evaluation would add richness to the data collection by providing direct information from the people with dementia, their carers and family about the real implications and effectiveness of the role which are often not captured in clinical tools.

While it was not clear which aspects of the support worker roles produced the most effective outcomes for people with dementia, their carers and families however some key areas of importance where identified. These areas of importance were drawn from studies that demonstrated significant outcomes and low risk of bias and identify the essential components for an ideal model. The essential components for key worker type support roles/interventions identified in this review provide guidance on how the key worker type support role can be best utilised to assist people with dementia living in the community and their carers. It is essential that a full description of the type of support model and the support provided in both the intervention and control groups is provided in any further research. These clear descriptions will also be useful for others looking to replicate the trial or implement the support model in other settings.

High quality randomised controlled trials of multi-disciplinary/collaborative holistic models of support are urgently needed. High-quality trials will also provide robust evidence in regard to cost-effectiveness and potential for cost savings of the support model as well as the emotional, physical and social benefits (quality of life, wellbeing, social support, reduction in symptoms and carer stress) for people with dementia, their carers and families.

### Implications for policy and practice

This review identified how dementia support workers are able to respond to the needs of people with dementia and their families throughout the course of the disease. Despite a paucity of high level evidence for the role the findings highlighted that dementia support workers have a unique potential to achieve person centred care and continuity of service through offering a single point of long term contact to the consumer. The needs of a person with dementia and their families vary over time and with these changes the need for assistance from health services also varies accordingly. The personalised nature of the support worker service mitigates the risk of this population reaching ‘crisis’ point which is when many have been observed to access services [6]. The inherent nature of the support worker service means the model/role can overcome issues such as fragmentation of services, poor service co-ordination and poor collaboration between providers by providing a ‘real person’ to assist with dementia related needs [6].

Despite limitations in the current evidence base for the support worker roles revealed by this systematic review there is enough evidence to warrant further exploration so that the essential components of the role can be incorporated into the design and funding of current and future community support services. The culmination of these findings has led us to recommend that the role be further examined so that greater evidence for the support worker models ability to contribute to the delivery of dementia care and the cost effectiveness of this role can be gathered.

### Strengths and limitations of the review

#### Strengths

Our extensive systematic mixed studies review of the international and national academic literature of models of support for community-dwelling people with dementia and their carers is the first to our knowledge to include both quantitative and qualitative studies and all models of support. Previous systematic reviews have focused mainly on the case management role, our review looks at all models of support for people with dementia, their families and carers. The investigation of both international and national models of support is also a key strength.

#### Limitations

A limitation to this review was that it was not possible to conduct a meta-analysis of results due to the heterogeneous nature of the articles and the interventions implemented. Furthermore, it is also possible that some studies were not identified as a result of the search terms that were used in each database.

## Conclusion

The strength of our synthesis of evidence is that it identifies the essential components of how key worker type support models could enhance current support models and how they can best be utilised to assist community-dwelling people with dementia and their carers. This review also reveals the poor evaluation design of many studies published to date: in the majority of cases, studies did not allow sufficient follow up time, many were not randomised and there was insufficient reporting in regard to blinding of outcome measures. Also as most studies were not registered there was an inability to determine if selective reporting occurred.

Studies that include a high quality evaluation of holistic, tailored models of support that identify which components of support produce the most valuable outcomes to assist people with dementia and their carers and families to continue to live meaningful lives are needed. There is also a need for a cost effectiveness evaluation of support worker roles.

## Abbreviations

CASP, critical appraisal skills programme; RCT, randomised controlled trial

## Additional file

Additional file 1: Appendices -Eligibility and critical appraisal instruments, studies assessed for eligibility, eligibility appraisal and included studies. (DOCX 71 kb)
