# Supplementary material for: What is the effectiveness of the support worker role for people with dementia and their carers? A systematic review
Source: BMC Health Serv Res. 2016 Jul 19;16:285. doi: 10.1186/s12913-016-1531-2 (PMC4950786; doi:10.1186/s12913-016-1531-2)
Supplement: Additional file 1: — Appendices -Eligibility and critical appraisal instruments, studies assessed for eligibility, eligibility appraisal and included studies. (DOCX 71 kb) [file 12913_2016_1531_MOESM1_ESM.docx]

## Appendix I: Eligibility assessment instrument

**Objective of SR:** To assess how the key worker role for people with dementia can best be utilised to assist community-dwelling people with dementia and their carers

**Participants** are community dwelling people who have dementia and their carers.

The **intervention** is the utilisation of a key worker (or link worker/case manager/support worker/clinical nurse consultant) skilled in assessment and support who provides ongoing assistance to older people with cognitive decline and their carers, ensuring continuity of care from the point of diagnosis.

**Reviewer _____________________________________Date_________________________________**

**Author_______________________________________Year____________Record number________**

| 1. Does the study involve utilisation or discussion of a key worker (or synonym) role?   Notes________________________________________   1. Do the participants have dementia or cognitive decline and/or are they carers of people with dementia/cognitive decline?   Notes________________________________________   1. Are the participants community dwelling/ living at home or are they carers of community dwelling people? | Yes No Unclear Not applicable ❑ ❑ ❑ ❑  Yes No Unclear Not applicable ❑ ❑ ❑ ❑  Yes No Unclear Not applicable ❑ ❑ ❑ ❑ |
| --- | --- |
| Notes________________________________________  Overall appraisal: Include ❑ Exclude ❑ Seek further info ❑ |  |
|  |  |
| Comments (including reason for exclusion) |  |

## Appendix II: Critical appraisal instruments

### Appraisal instrument for quantitative studies

| **Study design and validity** | **Question** | **How well was this criterion addressed?** |
| --- | --- | --- |
| Population | Was the source population well described? |  |
|  | Was the eligible population well described |  |
|  | Were relevant personal (prognostic) characteristics of the participants recorded? (e.g. characteristics to determine if person likely to be institutionalised in this case or more generally their outlook, such as co-morbidities) |  |
|  | How was the sample size determined? |  |
|  | Is the sample size calculation based on the primary outcome? |  |
|  | Is the study large enough to detect a clinically important difference? (e.g. a validated tool to measure score for depression decreases by say min of 5) |  |
| Exposure and comparison  groups | Are the exposure and comparison interventions well described and valid? |  |
|  | Was allocation to exposure and comparison groups randomised? |  |
|  | Was allocation concealed? |  |
|  | Were exposure and comparisons groups similar at baseline? If not, were differences adjusted for? |  |
|  | Were there any external interventions? (e.g. if participants in control/comparison group receive any external support that could cloud results) |  |
|  | Was contamination acceptably low? (e.g. if participants in comparison/control group receive intervention) |  |
|  | Was compliance in exposure and comparison groups adequate? (e.g. was there any reason(s)/barrier that meant intervention group or comparison group didn’t receive care described?) |  |
|  | Were all participants accounted for at the conclusion of the study? Was the proportion lost to follow up too high? Was it similar in both groups? |  |
| Outcomes | Were the outcome measures well described, reliable and valid? |  |
|  | Were participants, health staff and data collectors blind to group status? |  |
|  | Was outcome measurement complete |  |
|  | Were all important outcomes assessed? |  |
| Time | Was the follow-up time similar in the exposure and comparison groups? |  |
|  | Was the follow-up time meaningful? |  |
| **Results** | Were all participants analysed in the groups to which they were randomised (i.e. intention to treat analysis^[[1]](#footnote-1)^)? |  |
|  | Were appropriate statistical methods used? Were confounding variables adjusted for? |  |
|  | Could effect estimates (e.g. mean difference, risk ratio) be calculated? |  |
|  | How precise were the effect estimates (i.e. how narrow were the CIs)? |  |
|  | If no statistically significant effects detected, was the possibility of Type ll error discussed? (Type II error is failure to reject a false null hypothesis, represents a false negative. Example of type II error would be a clinical trial of a medical treatment failing to show that the treatment works when really it does) |  |
| **Applicability** |  |  |
| Generalisability | Was the source population for the study participants appropriate? |  |
|  | Were the study participants representative of the source population? |  |
|  | Were the study participants and setting well enough described to determine generalisability? |  |
| Feasibility and relevance | Is the intervention feasible and relevant to usual practice? |  |
|  | Was background management (i.e. comparison group management) relevant to usual practice |  |
| Applicability and potential harms | Were all important outcomes considered: benefits? Harms? Costs? |  |
|  | Are the benefits likely to be greater than any potential harms and costs (or vice versa)? In what target group(s)? |  |

**Source:** Questions drawn from Jackson R, Gate Framework checklist for randomised controlled trials and Schulz et al. (2010) CONSORT 2010 statement: Updated guidelines for reporting parallel group randomised trials. BMC Medicine, 8:18:1741-1715.

### Appraisal instrument for qualitative studies

| **Citation** | | |
| --- | --- | --- |
| **Question** | **Answer** | **Yes or no** |
| Did the paper describe an important clinical problem addressed via clearly formulated question? Did they answer the question they set out to? |  |  |
| Methodological approach |  |  |
| Were the methods appropriate for answering the research question/aim of study? |  |  |
| What was the setting? |  |  |
| Who were the participants? |  |  |
| How were the participants recruited? |  |  |
| Was recruitment appropriate for the aims? |  |  |
| Was there recruitment bias? |  |  |
| What was the researcher’s perspective and was this taken into account? |  |  |
| Interview schedule design |  |  |
| Data collection methods |  |  |
| Are data collection methods described in enough detail? (e.g. venue, mode, style of interview such as semi-structured) |  |  |
| Recording and transcription |  |  |
| Data analysis methods |  |  |
| Validity and reliability |  |  |
| Are the results credible? |  |  |
| What conclusions are drawn? |  |  |
| Are conclusions justified by the results? |  |  |
| Are the findings of the study transferable to other clinical settings? |  |  |

**Source:** Questions were drawn from Greenhalgh & Taylor (1997). How to read a paper: Papers that go beyond numbers

## Appendix III: Studies assessed for eligibility

### Academic Studies assessed for eligibility

| **Citation** | **Status** |
| --- | --- |
| 1. Abendstern M, Reilly S, Hughes J, Venables D, Challis D. Levels of integration and specialisation within professional community teams for people with dementia. International Journal of Geriatric Psychiatry. 2006 Jan; 21(1):77-85. PubMed PMID: 2006-01734-015. | Ineligible |
| 1. Adams KB, McClendon MJ. Early-stage cognitive impairment: A social work practice and research agenda. Families in Society. 2006 Oct-Dec; 87(4):590-600. PubMed PMID: 2007-07813-017. | Ineligible |
| 1. Aranda MP, Villa VM, Trejo L, Ramirez R, Ranney M. El Portal Latino Alzheimer's Project: Model Program for Latino Caregivers of Alzheimer's Disease-Affected People. Social Work. 2003 Apr; 48(2):259-71. PubMed PMID: 2003-00543-012. | Ineligible |
| 1. Asch-Goodkin J, Kaplan D. Alzheimer's dementia treatment recommendations. Patient Care. 2007; 41(1):8-. PubMed PMID: 2010282203. | Ineligible |
| 1. Atherly A, Kane RL, Smith MA: Older adults’ satisfaction with integrated capitated health and long-term care. Gerontologist 2004, 44(3):348-357. | Ineligible |
| 1. Austrom MG, Hartwell C, Moore P, Perkins AJ, Damush T, Unverzagt FW, et al. An integrated model of comprehensive care for people with Alzheimer's disease and their caregivers in a primary care setting. Dementia (14713012). 2006; 5(3):339-52. PubMed PMID: 2009278101. | Ineligible |
| 1. Banerjee S, Chan, J. (2008). Organisation of old age psychiatric services. Psychiatry 7(2): 49-54. | Ineligible |
| 1. Bass, D.M., Clark, P.A., Looman, W.J., McCarthy, C.A., & Eckert, S. (2003). The Cleveland Alzheimer’s managed care demonstration: Outcomes after 12 months of implementation. Gerontologist, 43, 73–85. | Appraised |
| 1. Beland F, Bergman H, Lebel P, Dallaire L, Fletcher J, Contandriopoulos AP, Tousignant P: Integrated services for frail elders (SIPA): A trial of a model for Canada. Canadian Journal on Aging 2006, 25(1):5-42. | Appraised |
| 1. Bierlein C, Hadjistavropoulos H, Bourgault-Fagnou M, Sagan M: A six-month profile of community case coordinated older adults. Can J Nurs Res 2006, 38(3):32-50. | Appraised |
| 1. Bird SR, Kurowski W, Dickman GK, Kronborg I. Integrated care facilitation for older patients with complex health care needs reduces hospital demand. Aust Health Rev. 2007;31(3):451-61. | Appraised |
| 1. Boughtwood D, Shanley C, Adams J, Santalucia Y, Kyriazopoulos H, Pond D, et al. Culturally and linguistically diverse (CALD) families dealing with dementia: An examination of the experiences and perceptions of multicultural community link workers. Journal of Cross-Cultural Gerontology. 2011 Dec; 26(4):365-77. PubMed PMID: 2011-27026-004. | Appraised |
| 1. Brodaty, H, Mittelman M, Gibson L, Seeher K, Burns A. 2009. The effects of counselling spouse caregivers of people with Alzheimer disease taking donepezil and of country of residence on rates of admission to nursing homes and mortality. Am J Geriatr Psychiatry 17: 734–743. | Appraised |
| 1. Burns, R., Nichols, L.O., Martindale-Adams, J., Graney, M.J., & Lummus, A. (2003). Primary care interventions for dementia caregivers: 2-year outcomes from the REACH study. Gerontologist, 43, 547–555. | Appraised |
| 1. Burton J, Hope KW. An exploration of the decision-making processes at the point of referral to an Admiral Nurse team. Journal of Psychiatric and Mental Health Nursing. 2005;12(3):359-64. PubMed PMID: 15876244. | Appraised |
| 1. [Callahan CM](http://www.ncbi.nlm.nih.gov/pubmed?term=Callahan%20CM%5BAuthor%5D&cauthor=true&cauthor_uid=16684985)1, [Boustani MA](http://www.ncbi.nlm.nih.gov/pubmed?term=Boustani%20MA%5BAuthor%5D&cauthor=true&cauthor_uid=16684985), [Unverzagt FW](http://www.ncbi.nlm.nih.gov/pubmed?term=Unverzagt%20FW%5BAuthor%5D&cauthor=true&cauthor_uid=16684985), [Austrom MG](http://www.ncbi.nlm.nih.gov/pubmed?term=Austrom%20MG%5BAuthor%5D&cauthor=true&cauthor_uid=16684985), [Damush TM](http://www.ncbi.nlm.nih.gov/pubmed?term=Damush%20TM%5BAuthor%5D&cauthor=true&cauthor_uid=16684985), [Perkins AJ](http://www.ncbi.nlm.nih.gov/pubmed?term=Perkins%20AJ%5BAuthor%5D&cauthor=true&cauthor_uid=16684985), [Fultz BA](http://www.ncbi.nlm.nih.gov/pubmed?term=Fultz%20BA%5BAuthor%5D&cauthor=true&cauthor_uid=16684985), [Hui SL](http://www.ncbi.nlm.nih.gov/pubmed?term=Hui%20SL%5BAuthor%5D&cauthor=true&cauthor_uid=16684985), [Counsell SR](http://www.ncbi.nlm.nih.gov/pubmed?term=Counsell%20SR%5BAuthor%5D&cauthor=true&cauthor_uid=16684985), [Hendrie HC](http://www.ncbi.nlm.nih.gov/pubmed?term=Hendrie%20HC%5BAuthor%5D&cauthor=true&cauthor_uid=16684985). (2006). Effectiveness of collaborative care for older adults with Alzheimer disease in primary care: a randomized controlled trial. [Journal of the American Medical Association.](http://www.ncbi.nlm.nih.gov/pubmed/16684985) May 10;295(18):2148-57. | Appraised |
| 1. Cherry, D. HCBS Can Keep People With Dementia at Home. Generations. 2012;36(1):83-90. PubMed PMID: 2011540315. | Ineligible |
| 1. Chien WT, Lee YM. A disease management program for families of persons in Hong Kong with dementia. Psychiatric services (Washington, DC. 2008 Apr;59:433-6. | Appraised |
| 1. Chien, W.T., Lee, I.Y.M. 2011. Randomised control trial of a dementia care program for families of home-resided older people with dementia. Journal of Advanced Nursing, 67(4), 774-787. | Appraised |
| 1. Chodosh J, Pearson ML, Connor KI, Vassar SD, Kaisey M, Lee ML, et al. A dementia care management intervention: which components improve quality? The American Journal of Managed Care. 2012;18(2):85-94. PubMed PMID: 22435836. | Appraised |
| 1. Clark P, Bass D, Looman W, McCarthy C, Eckert S. 2004. Outcomes for patients with dementia from the Cleveland Alzheimer’s managed care demonstration. Aging Ment Health 8(1): 245–51. | Appraised |
| 1. Cohen-Mansfield, J., et al. (2012). Knowledge of and Attitudes Toward Nonpharmacological Interventions for Treatment of Behavior Symptoms Associated With Dementia: A Comparison of Physicians, Psychologists, and Nurse Practitioners. Gerontologist 52(1): 34-45. | Ineligible |
| 1. Conley DM, Burket TL, Schumacher S, Lyons D, DeRosa SE, Schirm V. Implementing Geriatric Models of Care: A Role of the Gerontological Clinical Nurse Specialist-Part II. Geriatric Nursing. 2012;33(4):314-8. PubMed PMID: 2011651170. | Ineligible |
| 1. Connor K, McNeese-Smith D, Vickrey B. Classifying dementia care management activities: theory applied to practice. Communicating Nursing Research. 2007;40:399-. PubMed PMID: 2009905207. | Ineligible |
| 1. Derksen E, Vernooij-Dassen M, Scheltens P, Olde-Rikkert M. A model for disclosure of the diagnosis of dementia. Dementia (14713012). 2006;5(3):462-8. PubMed PMID: 2009278129. | Ineligible |
| 1. Dewing J, Traynor V. Admiral nursing competency project: practice development and action research. Journal of Clinical Nursing. 2005;14(6):695-703. PubMed PMID: 15946277. | Appraised |
| 1. Douris KR. Home-based primary care for dementia. Project ahead. Advance for Nurse Practitioners. 2003;11(1):50-2. PubMed PMID: 12630260. | Ineligible |
| 1. Downs M, Turner S, Bryans M, et al. 2006. Effectiveness of educational interventions in improving detection and management of dementia in primary care: A cluster randomized controlled study. BMJ 332: 692–695. | Ineligible |
| 1. Draper D, Brodaty H, Low LF. (2006) A tiered model of psychogeriatric service delivery: An evidence-based approach. International Journal of Geriatric Psychiatry 21: 645-653. | Ineligible |
| 1. Dröes R, Meiland FJM, de Lange J, Vernooij-Dassen MJF, van Tilburg W. The meeting centres support programme: an effective way of supporting people with dementia who live at home and their carers. Dementia (14713012). 2003;2(3):426-33. PubMed PMID: 2004062149. | Ineligible |
| 1. Duane F, Goeman DP, Beanland CJ and Koch SH. 2013. The role of the clinical nurse consultant dementia specialist: A qualitative evaluation. Dementia August 27, 2013 DOI: 10.1177/1471301213498759. | Appraised |
| 1. Duru OK, Ettner SL, Vassar SD, Chodosh J, Vickrey BG. Cost evaluation of a coordinated care management intervention for dementia. American Journal of Managed Care. 2009;15(8):521-8. PubMed PMID: 2010393211. | Appraised |
| 1. Dutton, R. (2009). Specialist community-based End of Life Dementia Care Nurse. Dementia (14713012) 8(3): 431-434. | Ineligible |
| 1. Eisdorfer, C., Czaja, S.J., Loewenstein, D.A., Rubert, M.P., Arguelles, S., Mitrani, V.B., et al. (2003). The effect of a family therapy and technology-based intervention on caregiver depression. Gerontologist, 43, 521–531. | Appraised |
| 1. Eloniemi-Sulkava U, Saarenheimo M, Laakkonen M, Pietilä M, Savikko N, Kautiainen H, et al. Family care as collaboration: effectiveness of a multicomponent support program for elderly couples with dementia. Randomized controlled intervention study. Journal of the American Geriatrics Society. 2009;57(12):2200-8. PubMed PMID: 2010504117. | Appraised |
| 1. Emilsson UM. Recognizing but not acknowledging: On using research information in social work with elderly people suffering from dementia. British Journal of Social Work. 2005 Dec;35(8):1393-409. PubMed PMID: 2005-15680-011. | Ineligible |
| 1. Etters L, Goodall D, Harrison BE. Caregiver burden among dementia patient caregivers: a review of the literature. Journal of the American Academy of Nurse Practitioners. 2008;20(8):423-8. PubMed PMID: 2010002571. | Ineligible |
| 1. Fischer LR, Green CA, Goodman MJ, Brody KK, Aickin M, Wei F, Phelps LW, Leutz W: Community-based care and risk of nursing home placement. Med Care 2003, 41(12):1407-1416 | Appraised |
| 1. Fortinsky RH, Kulldorff M, Kleppinger A, Kenyon-Pesce L. 2009. Dementia care consultation for family caregivers: collaborative model linking an Alzheimer’s association chapter with primary care physicians. Aging Ment Health 13: 162–170. | Appraised |
| 1. Galvin JE, Duda JE, Kaufer DI, Lippa CF, Taylor A, Zarit SH. Lewy body dementia: Caregiver burden and unmet needs. Alzheimer Disease and Associated Disorders. 2010 Apr-Jun;24(2):177-81. PubMed PMID: 2010-11453-011. | Ineligible |
| 1. Gaugler JE, Roth DL, Haley WE, Mittelman MS. 2008. Can counseling and support reduce burden and depressive symptoms in caregivers of people with Alzheimer’s disease during the transition to institutionalization? Results from the New York University caregiver intervention study. J Am Geriatr Soc 56: 421–428. | Appraised |
| 1. Ghiotti C. (2009) The Dementia End of Life Care project (DeLCaP) Supporting families caring for people with late stage dementia at home. Dementia 8(3): 349-361. | Ineligible |
| 1. Gilmour H, Gibson F, Campbell J. (2003). People with dementia in a rural community. Dementia 2(2): 245-263. | Ineligible |
| 1. Gould N. Integrating qualitative evidence in practice guideline development: Meeting the challenge of evidence-based practice for social work. Qualitative Social Work: Research and Practice. 2010 Mar;9(1):93-109. PubMed PMID: 2010-05234-009. | Ineligible |
| 1. Gould N. Guidelines across the health and social care divides: The example of the NICE-SCIE dementia guideline. International Review of Psychiatry. 2011 Aug;23(4):365-70. PubMed PMID: 2011-24798-009. | Ineligible |
| 1. Gould N, Kendall T. Developing the NICE/SCIE guidelines for dementia care: The challenges of enhancing the evidence base for social and health care. British Journal of Social Work. 2007 Apr;37(3):475-90. PubMed PMID: 2007-07666-006. | Ineligible |
| 1. Grabowski DC. The cost-effectiveness of noninstitutional long-term care services: review and synthesis of the most recent evidence. Medical Care Research and Review: MCRR. 2006;63(1):3-28. PubMed PMID: 16686071. | Ineligible |
| 1. Gravelle H, Dusheiko M, Sheaff R, Sargent P, Boaden R, Pickard S, Parker S, Roland M: Impact of case management (Evercare) on frail elderly patients: Controlled before and after analysis of quantitative outcome data. BMJ: British Medical Journal 2007, 334(7583):31. | Ineligible |
| 1. Gridley K, Brooks J, Glendinning C. Good practice in social care for disabled adults and older people with severe and complex needs: evidence from a scoping review. Health & Social Care In The Community. 2013. PubMed PMID: 23889999. | Ineligible |
| 1. Hall GR, Gallagher M, Dougherty J. Integrating roles for successful dementia management. Nurse Practitioner. 2009;34(11):35-41. PubMed PMID: 2010467917. Language: English. Entry Date: 20100101. Revision Date: 20100108. | Ineligible |
| 1. Hammar T, Perala ML, Rissanen P: The effects of integrated home care and discharge practice on functional ability and health-related quality of life: a cluster-randomised trial among home care patients. International Journal of Integrated Care 2007, 7:1-12. | Appraised |
| 1. He´bert, R., Levesque, L., Vezina, J., Lavoie, J.P., Ducharme, F., Gendron, C., et al. (2003). Efficacy of a psychoeducative group program for caregivers of demented persons living at home: A randomized controlled trial. Journal of Gerontology B: Psychological, 58, S58-S67 | Appraised |
| 1. Hibberd P. The Admiral Nurse Academy: a clinical academic pathway to support a specialist dementia nursing service. Quality in Ageing & Older Adults. 2011;12(2):95-102. PubMed PMID: 2011270477. | Ineligible |
| 1. Iliffe S, Wilcock J, Haworth D. Delivering psychosocial interventions for people with dementia in primary care: Jobs or skills? Dementia: The International Journal of Social Research and Practice. 2006 Aug;5(3):327-38. PubMed PMID: 2006-09614-003. | Ineligible |
| 1. Iliffe S, Wilcock J, Haworth D. (2006) Obstacles to shared care for patients with dementia: A qualitative study. Family Practice 23: 353-362. | Ineligible |
| 1. Iliffe S, Robinson L, Bamford C, Waugh A, Fox C, Livingston G, et al. Introducing case management for people with dementia in primary care: a mixed-methods study. British Journal of General Practice. 2014;64(628):e735-41. | Appraised |
| 1. Jansen APD, van Hout HPJ, Nijpels G, Rijmen F, Dröes R-M, Pot A-M, et al. Effectiveness of case management among older adults with early symptoms of dementia and their primary informal caregivers: a randomized clinical trial. International Journal of Nursing Studies. 2011;48(8):933-43. PubMed PMID: 21356537. | Appraised |
| 1. Johnson J, Hartle M. Social work and dementia care within adult day services. Dementia and social work practice: Research and interventions. New York, NY: Springer Publishing Co; US; 2007. p. 305-20. | Ineligible |
| 1. Jokinen N, Janicki MP, Keller SM, McCallion P, Force LT. Guidelines for Structuring Community Care and Supports for People With Intellectual Disabilities Affected by Dementia. Journal of Policy & Practice in Intellectual Disabilities. 2013;10(1):1-24. PubMed PMID: 2012045038. | Ineligible |
| 1. Kane RL, Homyak P, Bershadsky B, Flood S: Variations on a theme called PACE. Journals of Gerontology - Series a Biological Sciences and Medical Sciences 2006, 61(7):689-693. | Ineligible |
| 1. Keady J, Ashcroft-Simpson S, Halligan K, Williams S. Admiral nursing and the family care of a parent with dementia: Using autobiographical narrative as grounding for negotiated clinical practice and decision-making. Scandinavian Journal of Caring Sciences. 2007 Sep;21(3):345-53. PubMed PMID: 2007-12854-010. | Ineligible |
| 1. Koch T, Iliffe S, Manthorpe J, Stephens B, Fox C, Robinson L, et al. The potential of case management for people with dementia: A commentary. International Journal of Geriatric Psychiatry. 2012 Dec;27(12):1305-14. PubMed PMID: 2012-30189-011. | Ineligible |
| 1. Kontos T. Caring for persons with dementia in Australia. Dementia and social work practice: Research and interventions. New York, NY: Springer Publishing Co; US; 2007. p. 249-62. | Ineligible |
| 1. Lam LCW, Lee JSW, Chung JCC, Lau A, Woo J, Kwok TCY. A randomized controlled trial to examine the effectiveness of case management model for community dwelling older persons with mild dementia in Hong Kong. International Journal of Geriatric Psychiatry. 2010;25(4):395-402. PubMed PMID: 19606455. | Appraised |
| 1. Lombardo NE, Wu B, Chang K, Hohnstein JK. Model dementia care programs for Asian Americans. Dementia and social work practice: Research and interventions. New York, NY: Springer Publishing Co; US; 2007. p. 205-28. | Ineligible |
| 1. Low L-F, Yap M, Brodaty H. A systematic review of different models of home and community care services for older persons. BMC Health Services Research. 2011;11:93-. PubMed PMID: 21549010. | Ineligible |
| 1. Luchsinger J, Mittelman M, Mejia M, Silver S, Lucero RJ, Ramirez M, et al. The Northern Manhattan Caregiver Intervention Project: a randomised trial testing the effectiveness of a dementia caregiver intervention in Hispanics in New York City. BMJ Open. 2012;2(5). PubMed PMID: 22983877. | Ineligible |
| 1. MacNeil Vroomen J, Van Mierlo LD, van de Ven PM, Bosmans JE, van den Dungen P, Meiland FJM, et al. Comparing Dutch case management care models for people with dementia and their caregivers: The design of the COMPAS study. BMC Health Services Research. 2012;12:132-. PubMed PMID: 22640695. | Ineligible |
| 1. Mahoney, D.F., Tarlow, B.J., & Jones, R.N. (2003). Effects of an automated telephone support system on caregiver burden and anxiety: Findings from the REACH for TLC intervention study. Gerontologist, 43, 556–567. | Appraised |
| 1. Manthorpe J, Iliffe S. Changing the culture of social work support for people with early dementia. Australian Social Work. 2009 Jun;62(2):232-44. PubMed PMID: 2009-08828-008. | Ineligible |
| 1. Manthorpe J, Moriarty J. Models from other countries: Social work with people with dementia and their caregivers. Dementia and social work practice: Research and interventions. New York, NY: Springer Publishing Co; US; 2007. p. 229-47. | Ineligible |
| 1. Marek KD, Popejoy L, Petroski G, Rantz M: Nurse Care coordination in community-based long-term care. Journal of Nursing Scholarship 2006, 38(1):80-86. | Appraised |
| 1. McCollom P. When the diagnosis is Alzheimer's disease: case management as a resource. The Case Manager. 2004;15(6):64-6. PubMed PMID: 15557996. | Ineligible |
| 1. McDonald A, Heath B. Developing services for people with dementia. Working with Older People: Community Care Policy & Practice. 2009;13(3):18-21. PubMed PMID: 2010431377. | Ineligible |
| 1. McGhee G, Atkinson J. The carer/key worker relationship cycle: A theory of the reciprocal process. Journal of Psychiatric and Mental Health Nursing. 2010 May;17(4):312-8. PubMed PMID: 2010-08079-004. | Appraised |
| 1. Minkman MMN, Ligthart SA, Huijsman R. Integrated dementia care in The Netherlands: a multiple case study of case management programmes. Health & Social Care in the Community. 2009;17(5):485-94. PubMed PMID: 19694030. | Appraised |
| 1. Mittelman M, Haley W, Clay O, Roth D. 2006. Improving caregiver well-being delays nursing home placement of patients with Alzheimer Disease. Neurology 67: 1592–9. | Appraised |
| 1. Mittelman, M.S., Roth, D.L., Coon, D.W., & Haley, W.E. (2004). Sustained benefit of supportive intervention for depressive symptoms in caregivers of patients with Alzheimer’s disease. American Journal of Psychiatry, 161, 850–856. | Appraised |
| 1. Molony SL, Bouma R. The Care Manager Role in Person-Centered Care for People with Dementia. Generations. 2013;37(3):79-82. PubMed PMID: 2012390407. | Ineligible |
| 1. Morales-Asencio JM, Gonzalo-Jiménez E, Martin-Santos FJ, Morilla-Herrera JC, Celdráan-Mañas M, Carrasco AM, García-Arrabal JJ, Toral-López I: Effectiveness of a nurse-led case management home care model in Primary Health Care. A quasi-experimental, controlled, multi-centre study. BMC Health Services Research 2008, 8. | Appraised |
| 1. Morgan DG, Stewart NJ, Crossley M, D'Arcy C, Biem J, Kirk A, et al. Dementia Care in Rural and Remote Areas: The First Year of a CIHR New Emerging Team. CJNR: Canadian Journal of Nursing Research. 2005 Mar;37(1):177-82. PubMed PMID: 2005-03619-004. | Ineligible |
| 1. Nobili, A., Riva, E., Tettamanti, M., Lucca, U., Liscio, M., Petrucci, B., et al. (2004). The effect of a structured intervention on caregivers of patients with dementia and problem behaviors: A randomized controlled pilot study. Alzheimer Disease & Associated Disorder, 18, 75–82. | Appraised |
| 1. Onder G, Liperoti R, Bernabei R, Landi F: Case management, preventive strategies, and caregiver attitudes among older adults in home care: results of the ADHOC study. Journal of the American Medical Directors Association 2008, 9(5):337-341. | Appraised |
| 1. Onder G, Liperoti R, Soldato M, Carpenter I, Steel K, Bernabei R, Landi F: Case management and risk of nursing home admission for older adults in home care: results of the AgeD in HOme Care Study. J Am Geriatr Soc 2007, 55(3):439-444 | Appraised |
| 1. Peeters JM, Van Beek APA, Meerveld JHC, Spreeuwenberg PMM, Francke AL. Informal caregivers of persons with dementia, their use of and needs for specific professional support: a survey of the National Dementia Programme. BMC Nursing. 2010;9:9p. PubMed PMID: 2010728408. | Ineligible |
| 1. Pimouguet C, Lavaud T, Dartigues JF, Helmer C. Dementia case management effectiveness on health care costs and resource utilization: a systematic review of randomized controlled trials. The Journal of Nutrition, Health & Aging. 2010;14(8):669-76. PubMed PMID: 20922344. | Ineligible |
| 1. Pronsati MP. Deciphering dementia. Advance for NPs & PAs. 2012;3(9):8-. PubMed PMID: 2011671345. | Ineligible |
| 1. Quinn C, Clare L, McGuinness T, Woods RT. Negotiating the balance: The triadic relationship between spousal caregivers, people with dementia and Admiral Nurses. Dementia: The International Journal of Social Research and Practice. 2013 Sep;12(5):588-605. PubMed PMID: 2013-31309-006. | Appraised |
| 1. Ramsay M, Spence C. Models of dementia care in the community: a literature review. Mental Health Nursing. 2011;31(1):18-21. PubMed PMID: 2011027270. | Ineligible |
| 1. Rentz CA. Alzheimer's disease: an elusive thief. Nursing Management. 2008;39(6):33-8. PubMed PMID: 18536588. | Ineligible |
| 1. Robinson L, Iliffe S, Brayne C, Goodman C, Rait G, Manthorpe J, et al. Primary care and dementia: 2. long-term care at home: Psychosocial interventions, information provision, carer support and case management. International Journal of Geriatric Psychiatry. 2010 Jul;25(7):657-64. PubMed PMID: 2010-14115-001. | Ineligible |
| 1. Rothera I, Jones R, Harwood R, Avery AJ, Fisher K, James V, Shaw I, Waite J. (2008). An evaluation of a specialist multiagency home support service for older people with dementia using qualitative methods. International Journal of Geriatric Psychiatry 23: 65-72. | Appraised |
| 1. Samsi K, Manthorpe J, Nagendran T, Heath H. Challenges and expectations of the Mental Capacity Act 2005: An interview-based study of community-based specialist nurses working in dementia care. Journal of Clinical Nursing. 2012 Jun;21(11-12):1697-705. | Ineligible |
| 1. Sarna R, Thompson R. Admiral nurses' role in a dementia carers' information programme. Nursing Older People. 2008;20(9):30-4. PubMed PMID: 19048967. | Ineligible |
| 1. Schoenmakers B, Buntinx F, DeLepeleire J. Supporting the dementia family caregiver: The effect of home care intervention on general well-being. Aging & Mental Health. 2010 Jan;14(1):44-56. PubMed PMID: 2010-03099-004. | Ineligible |
| 1. Schulz, R, Burgio, L, Burns, R, Eisdorfer, C et al. (2003). Resources for Enhancing Alzheimer's Caregiver Health (REACH): Site-specific outcomes, and future directions. The Gerontologist; Aug 2003; 43, 4; pg. 514-20. | Ineligible |
| 1. Somme, D., Trouve, H., Dramé, M., Gagnon, D., Couturier, Y., Saint-Jean, O. (2012). Analysis of case management programs for patients with dementia: A systematic review. Alzheimer's & Dementia 8(5): 426-436. | Ineligible |
| 1. Specht J, Bossen A, Hall GR, Zimmerman B, Russell J. The effects of a dementia nurse care manager on improving caregiver outcomes. American Journal of Alzheimer's Disease and Other Dementias. 2009 Jun-Jul;24(3):193-207. PubMed PMID: 2009-10671-003. | Appraised |
| 1. St-Amant O, Ward-Griffin C, DeForge RT, Oudshoorn A, McWilliam C, Forbes D, et al. Making Care Decisions in Home-Based Dementia Care: Why Context Matters. Canadian Journal on Aging. 2012;31(4):423-34. PubMed PMID: 2011782670. | Ineligible |
| 1. Stevenson GS, Ewing H, Herschell J, Keith D. (2006). An enhanced assessment and support team (EAST) for dementing elders–review of a Scottish regional initiative. Journal of Mental Health 15(2): 251-258. | Appraised |
| 1. Tam-Tham H, Cepoiu-Martin M, Ronksley PE, Maxwell CJ, Hemmelgarn BR. Dementia case management and risk of long-term care placement: A systematic review and meta-analysis. International Journal of Geriatric Psychiatry. 2013 Sep;28(9):889-902. PubMed PMID: 2013-28263-002. | Ineligible |
| 1. Teri L, Gibbons LE, McCurry SM, et al. 2003. Exercise plus behavioral management in patients with Alzheimer disease: a randomized controlled trial. JAMA 290:2015–2022. | Appraised |
| 1. Trueland J. Listen and learn. Nursing Standard (Royal College Of Nursing (Great Britain): 1987). 2008;23(7):22-3. PubMed PMID: 18988578. | Ineligible |
| 1. Van Raak, A, Paulus A. (2008). The emergence of multidisciplinary teams for interagency service delivery in Europe: Is historical institutionalism wrong? Health Care Analysis. 16: 342-354. | Ineligible |
| 1. Verkade P-J, van Meijel B, Brink C, van Os-Medendorp H, Koekkoek B, Francke AL. Delphi research exploring essential components and preconditions for case management in people with dementia. BMC Geriatrics. 2010;10:54-. PubMed PMID: 20696035. | Appraised |
| 1. Vickrey BG, Mittman BS, Connor KI, Pearson ML, Della Penna RD, Ganiats TG, et al. The Effect of a Disease Management Intervention on Quality and Outcomes of Dementia Care: A Randomized, Controlled Trial. Annals of Internal Medicine. 2006;145(10):713-26. | Appraised |
| 1. Ward-Griffin C, Hall J, DeForge R, St-Amant O, McWilliam C, Oudshoorn A, et al. Dementia Home Care Resources: How Are We Managing? Journal of Aging Research. 2012:11p. PubMed PMID: 2011371081. | Ineligible |
| 1. Waugh, A., Austin, A., Manthorpe, J., Fox, C., Stephens, B., Robinson, L., Iliffe, S. (2013). Designing a complex intervention for dementia case management in primary care. BMC Family Practice 14: 101-101. | Ineligible |
| 1. Waugh F. Where does risk feature in community care practice with older people with dementia who live alone? Dementia (14713012). 2009;8(2):205-22. PubMed PMID: 2010319394. | Ineligible |
| 1. Weaver FM, Hickey EC, Hughes SL, Parker V, Fortunato D, Rose J, Cohen S, Robbins L, Orr W, Priefer B, Wieland D, Baskins J: Providing all-inclusive care for frail elderly veterans: Evaluation of three models of care. J Am Geriatr Soc 2008, 56(2):345-353. | Appraised |
| 1. Woods RT, Wills W, Higginson IJ, Hobbins J, Whitby M. Support in the community for people with dementia and their carers: a comparative outcome study of specialist mental health service interventions. International Journal Of Geriatric Psychiatry. 2003;18(4):298-307. PubMed PMID: 12673605. | Appraised |
| 1. Wray LO, Shulan MD, Toseland RW, Freeman KE, Vásquez BE, Gao J. 2010. The effect of telephone support groups on costs of care for veterans with dementia. Gerontologist 50: 623–631. | Appraised |

### Academic literature deemed ineligible

| **Citation** | **Status; is it eligible?** | **Reason for ineligibility** |
| --- | --- | --- |
| 1. Abendstern M, Reilly S, Hughes J, Venables D, Challis D. Levels of integration and specialisation within professional community teams for people with dementia. International Journal of Geriatric Psychiatry. 2006 Jan;21(1):77-85. | Ineligible | Does not involve a key worker/support worker model; is about integration and specialisation within professional teams. |
| 1. Adams KB, McClendon MJ. Early-stage cognitive impairment: A social work practice and research agenda. Families in Society. 2006 Oct-Dec;87(4):590-600. | Ineligible | Does not involve a key worker/support worker model; is about social work practice. |
| 1. Aranda MP, Villa VM, Trejo L, Ramirez R, Ranney M. El Portal Latino Alzheimer's Project: Model Program for Latino Caregivers of Alzheimer's Disease-Affected People. Social Work. 2003 Apr;48(2):259-71. | Ineligible | Does not involve a key worker model; the model used is inter-organisational community-based, collaborative. |
| 1. Asch-Goodkin J, Kaplan D. Alzheimer's dementia treatment recommendations. Patient Care. 2007;41(1):8-. | Ineligible | Does not involve a key worker/support worker model; summarises Alzheimer’s treatment recommendations. |
| 1. Atherly A, Kane RL, Smith MA: Older adults’ satisfaction with integrated capitated health and long-term care. Gerontologist 2004,44(3):348-357. | Ineligible | Does not evaluate a key worker/support worker model, paper describes the development of an instrument to evaluate satisfaction with care for older adults. |
| 1. Austrom MG, Hartwell C, Moore P, Perkins AJ, Damush T, Unverzagt FW, et al. An integrated model of comprehensive care for people with Alzheimer's disease and their caregivers in a primary care setting. Dementia (14713012). 2006;5(3):339-52. | Ineligible | Does not evaluate a key worker/support worker model; paper describes an integrated model of comprehensive care for people with Alzheimer’s Disease and their caregivers in a primary care setting. Article states that the results of the intervention are not yet available. |
| 1. Banerjee S, Chan, J. (2008). Organisation of old age psychiatric services. Psychiatry 7(2): 49-54. | Ineligible | Does not involve a key worker/support worker model; macroscopic analysis of the organisation of old age psychiatric services. |
| 1. Cherry, D. HCBS Can Keep People With Dementia at Home. Generations. 2012;36(1):83-90. | Ineligible | Does not involve a key worker/support worker model; describes various home and community based services to keep people with dementia home. |
| 1. Cohen-Mansfield, J., et al. (2012). Knowledge of and Attitudes Toward Nonpharmacological Interventions for Treatment of Behavior Symptoms Associated With Dementia: A Comparison of Physicians, Psychologists, and Nurse Practitioners. Gerontologist 52(1): 34-45. | Ineligible | Does not involve a key worker/support worker model, not community focused; explores the attitudes and beliefs of Physicians, Psychologists and Nurse Practitioners concerning the use of pharmacological versus non pharmacological interventions for behavioural problems in nursing home residents with dementia. |
| 1. Conley DM, Burket TL, Schumacher S, Lyons D, DeRosa SE, Schirm V. Implementing Geriatric Models of Care: A Role of the Gerontological Clinical Nurse Specialist-Part II. Geriatric Nursing. 2012;33(4):314-8. | Ineligible | Not community focused, discusses the role of a Gerontological Clinic Nurse Specialist in a hospital setting. |
| 1. Connor K, McNeese-Smith D, Vickrey B. Classifying dementia care management activities: theory applied to practice. Communicating Nursing Research. 007;40:399-. | Ineligible | Not eligible - conference abstract reporting on a thesis. |
| 1. Derksen E, Vernooij-Dassen M, Scheltens P, Olde-Rikkert M. A model for disclosure of the diagnosis of dementia. Dementia (14713012). 2006;5(3):462-8. | Ineligible | Does not involve a key worker/support worker model; summarises a model for disclosure of the diagnosis of dementia that uses accepted principles of bad news communication. |
| 1. Douris KR. Home-based primary care for dementia. Project ahead. Advance For Nurse Practitioners. 2003;11(1):50-2. | Ineligible | Does not evaluate a key worker/support worker model; describes the Advances in Home-Based Primary Care for End of Life in Advancing Dementia (AHEAD) project using case study examples |
| 1. Downs M, Turner S, Bryans M, et al. 2006. Effectiveness of educational interventions in improving detection and management of dementia in primary care: A cluster randomized controlled study. BMJ 332: 692–695. | Ineligible | Does not evaluate a key worker/support worker model, evaluates an educational program for health professionals to improve the diagnosis of dementia. |
| 1. Draper D, Brodaty H, Low LF. (2006) A tiered model of psychogeriatric service delivery: An evidence-based approach. International Journal of Geriatric Psychiatry 21: 645-653. | Ineligible | Does not involve a key worker/support worker model; describes a tiered model for comprehensive evidence based planning of service delivery for mental disorders in late life. |
| 1. Dröes R, Meiland FJM, de Lange J, Vernooij-Dassen MJF, van Tilburg W. The meeting centres support programme: an effective way of supporting people with dementia who live at home and their carers. Dementia (14713012). 2003;2(3):426-33. | Ineligible | Does not involve a key worker/support worker model; describes the Meeting Centres Support Programme that was run in socio-cultural community centres in the neighbourhood. |
| 1. Dutton, R. (2009). "Specialist community-based End of Life Dementia Care Nurse." Dementia (14713012) 8(3): 431-434. | Ineligible | Does not evaluate a key worker/support worker model; focuses on end of life care for people with dementia |
| 1. Emilsson UM. Recognizing but not acknowledging: On using research information in social work with elderly people suffering from dementia. British Journal of Social Work. 2005 Dec;35(8):1393-409. | Ineligible | Does not involve a key worker/support worker model; discusses how the results of research can be used to improve practice in social work with older people with dementia. |
| 1. Etters L, Goodall D, Harrison BE. Caregiver burden among dementia patient caregivers: a review of the literature. Journal of the American Academy of Nurse Practitioners. 2008;20(8):423-8. | Ineligible | Does not involve a key worker/support worker model; is concerned with caregiver burden among dementia patients. |
| 1. Galvin JE, Duda JE, Kaufer DI, Lippa CF, Taylor A, Zarit SH. Lewy body dementia: Caregiver burden and unmet needs. Alzheimer Disease and Associated Disorders. 2010 Apr-Jun;24(2):177-81. | Ineligible | Does not involve a key worker/support worker model; aims to determine unmet needs of Lewy Body Dementia caregivers. |
| 1. Ghiotti C. (2009) The Dementia End of Life Care project (DeLCaP) Supporting families caring for people with late stage dementia at home. Dementia 8(3): 349-361. | Ineligible | Does not involve a key worker/support worker model; describes the development of the Dementia End of Life Care Project. |
| 1. Gilmour H, Gibson F, Campbell J. (2003). People with dementia in a rural community. Dementia 2(2): 245-263. | Ineligible | Does not involve a key worker/support worker model; this descriptive, exploratory study focuses on prevalence rates and living circumstances of 435 people with dementia residing in rural Northern Ireland. |
| 1. Gould N. Integrating qualitative evidence in practice guideline development: Meeting the challenge of evidence-based practice for social work. Qualitative Social Work: Research and Practice. 2010 Mar;9(1):93-109. | Ineligible | Does not involve a key worker/support worker model; is concerned with integrating qualitative evidence in social work practice guideline development. |
| 1. Gould N. Guidelines across the health and social care divides: The example of the NICE-SCIE dementia guideline. International Review of Psychiatry. 2011 Aug;23(4):365-70. | Ineligible | Does not involve a key worker/support worker model; considers the development of the first integrated health and social care guideline for dementia care. |
| 1. Gould N, Kendall T. Developing the NICE/SCIE guidelines for dementia care: The challenges of enhancing the evidence base for social and health care. British Journal of Social Work. 2007 Apr;37(3):475-90. | Ineligible | Does not involve a key worker/support worker model; reports on the process of establishing the United Kingdom’s first joint health and social care evidence based guideline for dementia care. |
| 1. Grabowski DC. The cost-effectiveness of noninstitutional long-term care services: review and synthesis of the most recent evidence. Medical Care Research and Review: MCRR. 2006;63(1):3-28. | Ineligible | Does not involve a key worker/support worker model; is concerned with the general cost-effectiveness of non institutional long-term care services in the United States. |
| 1. Gravelle H, Dusheiko M, Sheaff R, Sargent P, Boaden R, Pickard S, Parker S,Roland M: Impact of case management (Evercare) on frail elderly patients: Controlled before and after analysis of quantitative outcome data. BMJ: British Medical Journal 2007, 334(7583):31. | Ineligible | Case management of frail elderly people introduced an additional range of services into primary care without any associated reduction in hospital admissions. Not specific to dementia. |
| 1. Gridley K, Brooks J, Glendinning C. Good practice in social care for disabled adults and older people with severe and complex needs: evidence from a scoping review. Health & Social Care In The Community. 2013. | Ineligible | Not eligible- Scoping Review of literature. |
| 1. Hall GR, Gallagher M, Dougherty J. Integrating roles for successful dementia management. Nurse Practitioner. 2009;34(11):35-41. | Ineligible | Does not involve a key worker/support worker model; is concerned with the clinical management of dementia patients by Nurse Practitioners. |
| 1. Hibberd P. The Admiral Nurse Academy: a clinical academic pathway to support a specialist dementia nursing service. Quality in Ageing & Older Adults. 2011;12(2):95-102. | Ineligible | Not a study. Does not evaluate a key worker/support worker model; paper provides an overview of the Admiral Nurse Academy that provides a web-based resource for the clinical and academic development of all admiral nurses. |
| 1. Iliffe S, Wilcock J, Haworth D. Delivering psychosocial interventions for people with dementia in primary care: Jobs or skills? Dementia: The International Journal of Social Research and Practice. 2006 Aug;5(3):327-38 | Ineligible | General Practiced based and does not involve a key worker/support worker model; is concerned with psychosocial interventions for people with dementia. |
| 1. Iliffe S, Wilcock J, Haworth D. (2006) Obstacles to shared care for patients with dementia: A qualitative study. Family Practice 23: 353-362. | Ineligible | Does not involve a key worker/support worker model; qualitative study which investigates the perceptions of specialists and generalists about the potential for shared care of people with dementia. |
| 1. Johnson J, Hartle M. Social work and dementia care within adult day services. Dementia and social work practice: Research and interventions. New York, NY: Springer Publishing Co; US; 2007. p. 305-20. | Ineligible | Book Chapter. Does not involve a key worker/support worker model; explores five specific social work roles for dementia care within the adult day setting. |
| 1. Jokinen N, Janicki MP, Keller SM, McCallion P, Force LT. Guidelines for Structuring Community Care and Supports for People With Intellectual Disabilities Affected by Dementia. Journal of Policy & Practice in Intellectual Disabilities. 2013;10(1):1-24. | Ineligible | Not a study; presents US guidelines for structuring community care/supports for people with intellectual disabilities affected by dementia that are drawn from research literature as well as clinical experiences and demonstrated best practices. |
| 1. Kane RL, Homyak P, Bershadsky B, Flood S: Variations on a theme called PACE. Journals of Gerontology - Series A Biological Sciences and Medical Sciences 2006, 61(7):689-693. | Ineligible | Does not evaluate a key worker/support worker model but an integrated care model operating in the adult day care setting. |
| 1. Keady J, Ashcroft-Simpson S, Halligan K, Williams S. Admiral nursing and the family care of a parent with dementia: Using autobiographical narrative as grounding for negotiated clinical practice and decision-making. Scandinavian Journal of Caring Sciences. 2007 Sep;21(3):345-53. | Ineligible | Does not evaluate a key worker/support worker model but rather explores the concept of using narrative approaches to therapy in care practice with families of people with dementia. |
| 1. Koch T, Iliffe S, Manthorpe J, Stephens B, Fox C, Robinson L, et al. The potential of case management for people with dementia: A commentary. International Journal of Geriatric Psychiatry. 2012 Dec;27(12):1305 | Ineligible | Not eligible- Systematic Review. |
| 1. Kontos T. Caring for persons with dementia in Australia. Dementia and social work practice: Research and interventions. New York, NY: Springer Publishing Co; US; 2007. p. 249-62. | Ineligible | Book Chapter. Does not involve a key worker/support worker model; outlines Australia’s dementia policy, service development and social work models of practice. |
| 1. Lombardo NE, Wu B, Chang K, Hohnstein JK. Model dementia care programs for Asian Americans. Dementia and social work practice: Research and interventions. New York, NY: Springer Publishing Co; US; 2007. p. 205-28. | Ineligible | Not eligible- Article was included in a book chapter that was published within time criteria but the actual article was from before 2003. |
| 1. Low L-F, Yap M, Brodaty H. A systematic review of different models of home and community care services for older persons. BMC Health Services Research. 2011;11:93-. | Ineligible | Not eligible- Systematic Review. |
| 1. Luchsinger J, Mittelman M, Mejia M, Silver S, Lucero RJ, Ramirez M, et al. The Northern Manhattan Caregiver Intervention Project: a randomised trial testing the effectiveness of a dementia caregiver intervention in Hispanics in New York City. BMJ Open. 2012;2(5). | Ineligible | Not eligible – protocol paper. |
| 1. MacNeil Vroomen J, Van Mierlo LD, van de Ven PM, Bosmans JE, van den Dungen P, Meiland FJM, et al. Comparing Dutch case management care models for people with dementia and their caregivers: The design of the COMPAS study. BMC Health Services Research. 2012;12:132-. | Ineligible. | Not eligible- study protocol paper. |
| 1. Manthorpe J, Iliffe S. Changing the culture of social work support for people with early dementia. Australian Social Work. 2009 Jun;62(2):232-44. | Ineligible | Not eligible- policy and practice review. Does not involve a key worker/support worker model; outlines the potential roles for social workers in their response to people with suspected and early dementia. |
| 1. Manthorpe J, Moriarty J. Models from other countries: Social work with people with dementia and their caregivers. Dementia and social work practice: Research and interventions. New York, NY: Springer Publishing Co; US; 2007. p. 229-47. | Ineligible | Does not involve a key worker/support worker model; outlines social work practice with people with dementia and their caregivers in various regions of the globe. |
| 1. McCollom P. When the diagnosis is Alzheimer's disease: case management as a resource. The Case Manager. 2004;15(6):64-6. | Ineligible | Does not discuss key worker/support worker models; describes a case study of the signs and symptoms of a man as his Alzheimer’s Disease progresses. |
| 1. McDonald A, Heath B. Developing services for people with dementia. Working with Older People: Community Care Policy & Practice. 2009;13(3):18-21. | Ineligible | Does not involve a key worker/support worker model; describes a service mapping exercise in three rural countries in England. |
| 1. Molony SL, Bouma R. The Care Manager Role in Person-Centered Care for People with Dementia. Generations. 2013;37(3):79-82. | Ineligible | Not eligible- not a study: describes the care manger role in person-centred care for people with dementia with implications for research and policy. |
| 1. Morgan DG, Stewart NJ, Crossley M, D'Arcy C, Biem J, Kirk A, et al. Dementia Care in Rural and Remote Areas: The First Year of a CIHR New Emerging Team. CJNR: Canadian Journal of Nursing Research. 2005 Mar;37(1):177-82. | Ineligible | Does not discuss a key worker/support worker model; describes the research programs developed by the Canadian Institutes of Health Research for dementia care in rural and remote areas. |
| 1. Peeters JM, Van Beek APA, Meerveld JHC, Spreeuwenberg PMM, Francke AL. Informal caregivers of persons with dementia, their use of and needs for specific professional support: a survey of the National Dementia Programme. BMC Nursing. 2010;9:9p. | Ineligible | Does not discuss a key worker/support worker model; analyses informal caregivers use of and needs for specific professional support. |
| 1. Pimouguet C, Lavaud T, Dartigues JF, Helmer C. Dementia case management effectiveness on health care costs and resource utilization: a systematic review of randomized controlled trials. The Journal Of Nutrition, Health & Aging. 2010;14(8):669-76. | Ineligible | Not eligible- Systematic Review. |
| 1. Pronsati MP. Deciphering dementia. Advance for NPs & PAs. 2012;3(9):8-. | Ineligible | Does not discuss a key worker/support worker model; column/editorial that introduces the issue of dementia management. |
| 1. Ramsay M, Spence C. Models of dementia care in the community: a literature review. Mental Health Nursing. 2011;31(1):18-21. | Ineligible | Not eligible- literature review. Summarises various models of dementia care in the community. |
| 1. Rentz CA. Alzheimer's disease: an elusive thief. Nursing Management. 2008;39(6):33-8. | Ineligible | Does not discuss a key worker/support worker model; describes the course/progression of Alzheimer’s Disease and the implications for nurses. |
| 1. Robinson L, Iliffe S, Brayne C, Goodman C, Rait G, Manthorpe J, et al. Primary care and dementia: 2. long-term care at home: Psychosocial interventions, information provision, carer support and case management. International Journal of Geriatric Psychiatry. 2010 Jul;25(7):657-64. | Ineligible | Not eligible – narrative review. Discusses the role of the primary care physicians in the long-term care of people with dementia living at home. |
| 1. Samsi K, Manthorpe J, Nagendran T, Heath H. Challenges and expectations of the Mental Capacity Act 2005: An interview-based study of community-based specialist nurses working in dementia care. Journal of Clinical Nursing. 2012 Jun;21(11-12):1697-705. | Ineligible | Not eligible – not a study/evaluation. Describes the Medical Capacity Act and barriers to Admiral Nurses using it with their clients. |
| 1. Sarna R, Thompson R. Admiral nurses' role in a dementia carers' information programme. Nursing Older People. 2008;20(9):30-4. | Ineligible. | Not eligible – not a study/evaluation. Describes delivery of a carer’s information programme which aims to provide educational and emotional support to carers of people with dementia. |
| 1. Schoenmakers B, Buntinx F, DeLepeleire J. Supporting the dementia family caregiver: The effect of home care intervention on general well-being. Aging & Mental Health. 2010 Jan;14(1):44-56. | Ineligible | Not eligible – Systematic Review. |
| 1. Schulz, R, Burgio, L, Burns, R, Eisdorfer, C et al. (2003). Resources for Enhancing Alzheimer's Caregiver Health (REACH): Site-specific outcomes, and future directions. The Gerontologist; Aug 2003; 43, 4; pg. 514-20. | Ineligible | Not eligible- summaries outcomes from individual REACH studies which are already included in the review |
| 1. Somme, D., Trouve, H., Dramé, M., Gagnon, D., Couturier, Y., Saint-Jean, O. (2012). Analysis of case management programs for patients with dementia: A systematic review. Alzheimer's & Dementia 8(5): 426-436. | Ineligible | Not eligible- Systematic Review. |
| 1. St-Amant O, Ward-Griffin C, DeForge RT, Oudshoorn A, McWilliam C, Forbes D, et al. Making Care Decisions in Home-Based Dementia Care: Why Context Matters. Canadian Journal on Aging. 2012;31(4):423-34. | Ineligible | Does not discuss a key worker/support worker model; is an ethnographic study examining how the context of the home care system shapes decision making for people with dementia and their caregivers. |
| 1. Tam-Tham H, Cepoiu-Martin M, Ronksley PE, Maxwell CJ, Hemmelgarn BR. Dementia case management and risk of long-term care placement: A systematic review and meta-analysis. International Journal of Geriatric Psychiatry. 2013 Sep;28(9):889-902. | Ineligible | Not eligible- Systematic Review. |
| 1. Trueland J. Listen and learn. Nursing Standard (Royal College Of Nursing (Great Britain): 1987). 2008; 23(7):22-3. | Ineligible | Does not discuss a key worker/support worker model; is a short article describing a course set up in Scotland to educate nurses in dementia care. |
| 1. Van Raak, A, Paulus A. (2008). The emergence of multidisciplinary teams for interagency service delivery in Europe: Is historical institutionalism wrong? Health Care Analysis. 16: 342-354. | Ineligible | Does not discuss a key worker/support worker model; paper is a macroscopic level examination of the emergence of multi-disciplinary teams for people of all chronic illnesses. |
| 1. Ward-Griffin C, Hall J, DeForge R, St-Amant O, McWilliam C, Oudshoorn A, et al. Dementia Home Care Resources: How Are We Managing? Journal of Aging Research. 2012:11p. | Ineligible | Does not discuss a key worker/support worker model; the critical ethnographic study aims to develop a better understanding of home-based dementia care within a sociocultural context. |
| 1. Waugh, A., Austin, A., Manthorpe, J., Fox, C., Stephens, B., Robinson, L., Iliffe, S. (2013). Designing a complex intervention for dementia case management in primary care. BMC Family Practice 14: 101-101. | Ineligible | Does not evaluate a key/support worker model; describes the generic skills and personal attributes needed for practice nurse taking up the case manager role in their workplaces and for social workers in general practice teams |
| 1. Waugh F. Where does risk feature in community care practice with older people with dementia who live alone? Dementia (14713012). 2009; 8(2):205-22. | Ineligible | Does not discuss a key worker/support worker model; reports on five practitioner’s perspectives of what were the key elements of practice in community care for older persons with dementia who live alone. |

## Appendix IV: Table of included studies

| **Citation** | **Status** |
| --- | --- |
| 1. Bass, D.M., Clark, P.A., Looman, W.J., McCarthy, C.A., & Eckert, S. (2003). The Cleveland Alzheimer’s managed care demonstration: Outcomes after 12 months of implementation. Gerontologist, 43, 73–85. | Appraised |
| 1. Beland F, Bergman H, Lebel P, Dallaire L, Fletcher J, Contandriopoulos AP, Tousignant P: Integrated services for frail elders (SIPA): A trial of a model for Canada. Canadian Journal on Aging 2006, 25(1):5-42. | Appraised |
| 1. Bierlein C, Hadjistavropoulos H, Bourgault-Fagnou M, Sagan M: A six-month profile of community case coordinated older adults. Can J Nurs Res 2006, 38(3):32-50. | Appraised |
| 1. Bird SR, Kurowski W, Dickman GK, Kronborg I: Integrated care facilitation for older patients with complex health care needs reduces hospital demand. Australian health review: a publication of the Australian Hospital Association 2007, 31(3). | Appraised |
| 1. Boughtwood D, Shanley C, Adams J, Santalucia Y, Kyriazopoulos H, Pond D, et al. Culturally and linguistically diverse (CALD) families dealing with dementia: An examination of the experiences and perceptions of multicultural community link workers. Journal of Cross-Cultural Gerontology. 2011 Dec;26(4):365-77. | Appraised |
| 1. Brodaty, H, Mittelman M, Gibson L, Seeher K, Burns A. 2009. The effects of counselling spouse caregivers of people with Alzheimer disease taking donepezil and of country of residence on rates of admission to nursing homes and mortality. Am J Geriatr Psychiatry 17: 734–743. | Appraised |
| 1. Brown, J., et al. (2012). Service and Support Requirements of People with Younger Onset Dementia and their Families, NSW Department of Family and Community Services, Ageing, Disability and Home Care, Sydney. | Appraised |
| 1. Burns, R., Nichols, L.O., Martindale-Adams, J., Graney, M.J., & Lummus, A. (2003). Primary care interventions for dementia caregivers: 2-year outcomes from the REACH study. Gerontologist, 43, 547–555. | Appraised |
| 1. Burton J, Hope KW. An exploration of the decision-making processes at the point of referral to an Admiral Nurse team. Journal Of Psychiatric And Mental Health Nursing. 2005;12(3):359-64. | Appraised |
| 1. [Callahan CM](http://www.ncbi.nlm.nih.gov/pubmed?term=Callahan%20CM%5BAuthor%5D&cauthor=true&cauthor_uid=16684985)1, [Boustani MA](http://www.ncbi.nlm.nih.gov/pubmed?term=Boustani%20MA%5BAuthor%5D&cauthor=true&cauthor_uid=16684985), [Unverzagt FW](http://www.ncbi.nlm.nih.gov/pubmed?term=Unverzagt%20FW%5BAuthor%5D&cauthor=true&cauthor_uid=16684985), [Austrom MG](http://www.ncbi.nlm.nih.gov/pubmed?term=Austrom%20MG%5BAuthor%5D&cauthor=true&cauthor_uid=16684985), [Damush TM](http://www.ncbi.nlm.nih.gov/pubmed?term=Damush%20TM%5BAuthor%5D&cauthor=true&cauthor_uid=16684985), [Perkins AJ](http://www.ncbi.nlm.nih.gov/pubmed?term=Perkins%20AJ%5BAuthor%5D&cauthor=true&cauthor_uid=16684985), [Fultz BA](http://www.ncbi.nlm.nih.gov/pubmed?term=Fultz%20BA%5BAuthor%5D&cauthor=true&cauthor_uid=16684985), [Hui SL](http://www.ncbi.nlm.nih.gov/pubmed?term=Hui%20SL%5BAuthor%5D&cauthor=true&cauthor_uid=16684985), [Counsell SR](http://www.ncbi.nlm.nih.gov/pubmed?term=Counsell%20SR%5BAuthor%5D&cauthor=true&cauthor_uid=16684985), [Hendrie HC](http://www.ncbi.nlm.nih.gov/pubmed?term=Hendrie%20HC%5BAuthor%5D&cauthor=true&cauthor_uid=16684985). (2006). Effectiveness of collaborative care for older adults with Alzheimer disease in primary care: a randomized controlled trial. [Journal of the American Medical Association.](http://www.ncbi.nlm.nih.gov/pubmed/16684985) May 10;295(18):2148-57. | Appraised |
| 1. Cartmell, N. and D. Bardsley (2011). An Evaluation of Dementia Support Worker Roles, South West Dementia Partnership. | Appraised |
| 1. Chien WT, Lee YM. A disease management program for families of persons in Hong Kong with dementia. Psychiatric services (Washington, DC. 2008 Apr;59:433-6. | Appraised |
| 1. Chien, W.T., Lee, I.Y.M. 2011. Randomised control trial of a dementia care program for families of home-resided older people with dementia. Journal of Advanced Nursing, 67(4), 774-787. | Appraised |
| 1. Chodosh J, Pearson ML, Connor KI, Vassar SD, Kaisey M, Lee ML, et al. A dementia care management intervention: which components improve quality? The American Journal Of Managed Care. 2012;18(2):85-94. | Appraised |
| 1. Clare, L., et al. (2005). Carers' experiences of the Admiral Nurse Service: How well are Admiral Nurse Service Standards 1, 2 and 3 being met? London, Dementia Services Development Centre, University of Wales Bangor. | Appraised |
| 1. Clark P, Bass D, Looman W, McCarthy C, Eckert S. 2004. Outcomes for patients with dementia from the Cleveland Alzheimer’s managed care demonstration. Aging Ment Health 8(1): 245–51. | Appraised |
| 1. Clarke, C. L., et al. (2013). HEALTHBRIDGE: The National Evaluation of Peer Support Networks and Dementia Advisers in implementation of the National Dementia Strategy for England, Department of Health Policy Research Programme Project, Department of Health. | Appraised |
| 1. Dewing J, Traynor V. Admiral nursing competency project: practice development and action research. Journal Of Clinical Nursing. 2005;14(6):695-703. | Appraised |
| 1. Duane F, Goeman DP, Beanland CJ and Koch SH. 2013. The role of the clinical nurse consultant dementia specialist: A qualitative evaluation. Dementia, August 27, 2013 1471301213498759. | Appraised |
| 1. Duru OK, Ettner SL, Vassar SD, Chodosh J, Vickrey BG. Cost evaluation of a coordinated care management intervention for dementia. American Journal of Managed Care. 2009;15(8):521-8. | Appraised |
| 1. Edgecombe, K., et al. (2009). Dementia Care Evaluation, Veterans Affairs Canada, Canada. | Appraised |
| 1. Eisdorfer, C., Czaja, S.J., Loewenstein, D.A., Rubert, M.P., Arguelles, S., Mitrani, V.B., et al. (2003). The effect of a family therapy and technology-based intervention on caregiver depression. Gerontologist, 43, 521–531. | Appraised |
| 1. Eloniemi-Sulkava U, Saarenheimo M, Laakkonen M, Pietilä M, Savikko N, Kautiainen H, et al. Family care as collaboration: effectiveness of a multicomponent support program for elderly couples with dementia. Randomized controlled intervention study. Journal of the American Geriatrics Society. 2009;57(12):2200-8. | Appraised |
| 1. Fischer LR, Green CA, Goodman MJ, Brody KK, Aickin M, Wei F, Phelps LW, Leutz W: Community-based care and risk of nursing home placement. Med Care 2003, 41(12):1407-1416 | Appraised |
| 1. Fortinsky RH, Kulldorff M, Kleppinger A, Kenyon-Pesce L. 2009. Dementia care consultation for family caregivers: collaborative model linking an Alzheimer’s association chapter with primary care physicians. Aging Ment Health 13: 162–170. | Appraised |
| 1. Frances Morton Consulting (2010). Dementia Care: Diversity, Access, Coordination Final Report, Alzheimer Society of Toronto. | Appraised |
| 1. Gaugler JE, Roth DL, Haley WE, Mittelman MS. 2008. Can counseling and support reduce burden and depressive symptoms in caregivers of people with Alzheimer’s disease during the transition to institutionalization? Results from the New York University caregiver intervention study. J Am Geriatr Soc 56: 421–428. | Appraised |
| 1. Hammar T, Perala ML, Rissanen P: The effects of integrated home care and discharge practice on functional ability and health-related quality of life: a cluster-randomised trial among home care patients. International Journal of Integrated Care 2007, 7:1-12. | Appraised |
| 1. He´bert, R., Levesque, L., Vezina, J., Lavoie, J.P., Ducharme, F., Gendron, C., et al. (2003). Efficacy of a psychoeducative group program for caregivers of demented persons living at home: A randomized controlled trial. Journal of Gerontology B: Psychological, 58, S58-S67 | Appraised |
| 1. Iliffe S, Robinson L, Bamford C, Waugh A, Fox C, Livingston G, et al. Introducing case management for people with dementia in primary care: a mixed-methods study. British Journal of General Practice. 2014;64(628):e735-41. | Appraised |
| 1. Jansen APD, van Hout HPJ, Nijpels G, Rijmen F, Dröes R-M, Pot A-M, et al. Effectiveness of case management among older adults with early symptoms of dementia and their primary informal caregivers: a randomized clinical trial. International Journal Of Nursing Studies. 2011;48(8):933-43. | Appraised |
| 1. King, A., et al. (2011). Evaluation of a Gerontology Nurse Specialist in Primary Health Care: Case Finding, Care Coordination and Service Integration for At-Risk Older People, Health Workforce New Zealand, Auckland, New Zealand. | Appraised |
| 1. La Fontaine, J., et al. (2011). Evaluation Report Early Intervention Dementia Service Worcestershire, Association for Dementia Studies University of Worcester, Worcester, United Kingdom. | Appraised |
| 1. Lam LCW, Lee JSW, Chung JCC, Lau A, Woo J, Kwok TCY. A randomized controlled trial to examine the effectiveness of case management model for community dwelling older persons with mild dementia in Hong Kong. International Journal Of Geriatric Psychiatry. 2010;25(4):395-402. | Appraised |
| 1. Mahoney, D.F., Tarlow, B.J., & Jones, R.N. (2003). Effects of an automated telephone support system on caregiver burden and anxiety: Findings from the REACH for TLC intervention study. Gerontologist, 43, 556–567. | Appraised |
| 1. Marek KD, Popejoy L, Petroski G, Rantz M: Nurse care coordination in community-based long-term care. Journal of Nursing Scholarship 2006, 38(1):80-86. | Appraised |
| 1. McAiney, C. A., et al. (2010). First Link Demonstration Project: Final Evaluation Report, Alzheimer Society of Ontario, Toronto, Ontario, Canada. | Appraised |
| 1. McGhee G, Atkinson J. The carer/key worker relationship cycle: A theory of the reciprocal process. Journal of Psychiatric and Mental Health Nursing. 2010 May;17(4):312-8. | Appraised |
| 1. Minkman MMN, Ligthart SA, Huijsman R. Integrated dementia care in The Netherlands: a multiple case study of case management programmes. Health & Social Care In The Community. 2009;17(5):485-94. | Appraised |
| 1. Mittelman M, Haley W, Clay O, Roth D. 2006. Improving caregiver well-being delays nursing home placement of patients with Alzheimer Disease. Neurology 67: 1592–9. | Appraised |
| 1. Mittelman, M.S., Roth, D.L., Coon, D.W., & Haley, W.E. (2004). Sustained benefit of supportive intervention for depressive symptoms in caregivers of patients with Alzheimer’s disease. American Journal of Psychiatry, 161, 850–856. | Appraised |
| 1. Moore, K. and E. Renehan (2011). Evaluation of Linking Lives Pilot: Supporting younger people with dementia Final Report for Alzheimer's Australia Victoria, National Ageing Research Institute, Victoria, Australia. | Appraised |
| 1. Morales-Asencio JM, Gonzalo-Jiménez E, Martin-Santos FJ, Morilla-Herrera JC, Celdráan-Mañas M, Carrasco AM, García-Arrabal JJ, Toral-López I: Effectiveness of a nurse-led case management home care model in Primary Health Care. A quasi-experimental, controlled, multi-centre study. BMC Health Services Research 2008, 8. | Appraised |
| 1. Nobili, A., Riva, E., Tettamanti, M., Lucca, U., Liscio, M., Petrucci, B., et al. (2004). The effect of a structured intervention on caregivers of patients with dementia and problem behaviors: A randomized controlled pilot study. Alzheimer Disease & Associated Disorder, 18, 75–82. | Appraised |
| 1. Onder G, Liperoti R, Bernabei R, Landi F: Case management, preventive strategies, and caregiver attitudes among older adults in home care: results of the ADHOC study. Journal of the American Medical Directors Association 2008, 9(5):337-341. | Appraised |
| 1. Onder G, Liperoti R, Soldato M, Carpenter I, Steel K, Bernabei R, Landi F: Case management and risk of nursing home admission for older adults in home care: results of the AgeD in HOme Care Study. J Am Geriatr Soc 2007, 55(3):439-444 | Appraised |
| 1. Quinn C, Clare L, McGuinness T, Woods RT. Negotiating the balance: The triadic relationship between spousal caregivers, people with dementia and Admiral Nurses. Dementia: The International Journal of Social Research and Practice. 2013 Sep;12(5):588-605. | Appraised |
| 1. Rothera I, Jones R, Harwood R, Avery AJ, Fisher K, James V, Shaw I, Waite J. (2008). An evaluation of a specialist multiagency home support service for older people with dementia using qualitative methods. International Journal of Geriatric Psychiatry 23: 65-72. | Appraised |
| 1. Specht J, Bossen A, Hall GR, Zimmerman B, Russell J. The effects of a dementia nurse care manager on improving caregiver outcomes. American Journal of Alzheimer's Disease and Other Dementias. 2009 Jun-Jul;24(3):193-207. | Appraised |
| 1. Stevenson GS, Ewing H, Herschell J, Keith D. (2006). An enhanced assessment and support team (EAST) for dementing elders–review of a Scottish regional initiative. Journal of Mental Health 15(2): 251-258. | Appraised |
| 1. Tam-Tham, H. (2013). A comparative study of dementia-related service provision and configuration in Canada. Faculty of Graduate Studies, Department of Community Health Sciences University of Calgary, Alberta, Canada. Degree of Master of Science. | Appraised |
| 1. Teri L, Gibbons LE, McCurry SM, et al. 2003. Exercise plus behavioral management in patients with Alzheimer disease: a randomized controlled trial. JAMA 290:2015–2022. | Appraised |
| 1. van den Bosch, L. (2009). The application of FO/BO and modularity theory on the design of case management in the context of dementia care. Department Organization & Strategy, Faculty of Economics and Business Administration (FEB), Tilburg University. | Appraised |
| 1. Verkade P-J, van Meijel B, Brink C, van Os-Medendorp H, Koekkoek B, Francke AL. Delphi research exploring essential components and preconditions for case management in people with dementia. BMC Geriatrics. 2010;10:54-. | Appraised |
| 1. Vickrey BG, Mittman BS, Connor KI, Pearson ML, Della Penna RD, Ganiats TG, et al. The Effect of a Disease Management Intervention on Quality and Outcomes of Dementia Care: A Randomized, Controlled Trial. Annals of Internal Medicine. 2006;145(10):713-26. | Appraised |
| 1. Weaver FM, Hickey EC, Hughes SL, Parker V, Fortunato D, Rose J, Cohen S, Robbins L, Orr W, Priefer B, Wieland D, Baskins J: Providing all-inclusive care for frail elderly veterans: Evaluation of three models of care. J Am Geriatr Soc 2008, 56(2):345-353. | Appraised |
| 1. Woods RT, Wills W, Higginson IJ, Hobbins J, Whitby M. Support in the community for people with dementia and their carers: a comparative outcome study of specialist mental health service interventions. International Journal Of Geriatric Psychiatry. 2003;18(4):298-307. | Appraised |
| 1. Wray LO, Shulan MD, Toseland RW, Freeman KE, Vásquez BE, Gao J. 2010. The effect of telephone support groups on costs of care for veterans with dementia. Gerontologist 50: 623–631. | Appraised |

1. [↑](#footnote-ref-1)
